# Traditional Chinese medicine syndrome patterns and associated factors in adults with type 2 diabetes and metabolic syndrome: a data-driven analysis

**DOI:** 10.3389/fendo.2026.1843491

**Published:** 2026-06-17

**Authors:** Jialing Zhang, Zhilin Lin, Shuyan Zhong, Minxia Ma, Hoi Ki Wong, Liz Sin Li, Kenneth Ka Hei Lo, Zhaoxiang Bian

**Affiliations:** 1 Vincent V.C. Woo Chinese Medicine Clinical Research Institute, School of Chinese Medicine, Hong Kong Baptist University, Hong Kong, Hong Kong SAR, China; 2 School of Chinese Medicine, Hong Kong Baptist University, Hong Kong, Hong Kong SAR, China; 3 Department of Food Science and Nutrition, The Hong Kong Polytechnic University, Hong Kong, Hong Kong SAR, China; 4 Chinese EQUATOR Centre, Hong Kong Baptist University, Hong Kong, Hong Kong SAR, China; 5 Centre for Chinese Herbal Medicine Drug Development, Hong Kong Baptist University, Hong Kong, Hong Kong SAR, China

**Keywords:** latent class analysis, metabolic syndrome, principal component analysis, syndrome differentiation, traditional Chinese medicine, type 2 diabetes

## Abstract

**Background:**

Syndrome differentiation is fundamental to traditional Chinese medicine (TCM) diagnosis and treatment, yet its application is complicated by population heterogeneity and disease phenotypes. Data-driven methods to patient stratification offer a pathway to refine syndromic classification beyond expert consensus. This study aimed to identify and characterize TCM syndrome-based patient subgroups and their associated factors in a Hong Kong cohort with comorbid type 2 diabetes mellitus (T2DM) and metabolic syndrome (MetS).

**Methods:**

This cross-sectional study included 505 adults with comorbid T2DM and MetS in Hong Kong. Data on TCM symptoms, clinical profiles, and patient-reported outcomes were collected. To identify patient subgroups, we applied principal component analysis (PCA) and cluster analysis to derive syndrome constructs, and latent class analysis (LCA) to identify latent patient subgroups based on symptom patterns. Multinomial logistic regression was used to explore factors associated with the derived subgroups.

**Results:**

Complementary data-driven approaches identified distinct patient subgroups based on TCM syndrome patterns. PCA derived 5 symptom-based syndromes: Spleen and Kidney Yang Deficiency (17.62%), Qi and Yin Deficiency (22.97%), Kidney Essence Deficiency (10.89%), Yin and Yang Deficiency (22.97%), and Phlegm and Blood Stasis (25.54%). LCA identified 5 latent patient subgroups: Liver Depression and Spleen Deficiency (14.46%), Liver Depression and Spleen Deficiency with Qi and Yin Deficiency (14.46%), Liver and Kidney Yin Deficiency (36.24%), Qi and Yin Deficiency with Phlegm-Blood Stasis (13.27%), and Phlegm and Blood Stasis (21.58%). Multinomial regression indicated that syndrome patterns were significantly associated with multiple factors (all *P* < 0.05), including sociodemographic characteristics (age, gender, monthly household income), clinical parameters (hypertension, triglyceride levels, basal metabolic rate), lifestyle behaviors (alcohol and caffeine consumption), and patient-reported outcomes (fatigue severity, Pittsburgh Sleep Quality Index score, Audit of diabetes-dependent quality of Life weighted score, and energy intake).

**Conclusion:**

This study demonstrates that complementary data-driven methods, specifically PCA and LCA, can effectively map the heterogeneous landscape of TCM syndromes in patients with comorbid T2DM and MetS. The analysis validates core constructs, including Phlegm and Blood Stasis, and links deficiency syndromes to severe fatigue and poor sleep. Future TCM syndrome research may benefit from prioritizing these empirically derived, multidimensional classifications to inform the development of personalized management strategies.

**Clinical trial registration:**

## Introduction

Diabetes mellitus (DM) has emerged as a critical global public health crisis, with 529 million individuals affected and an age-standardized prevalence of 6.1% worldwide in 2021 (1). Type 2 diabetes mellitus (T2DM) comprises 96% of diabetes and is a leading cause of global morbidity, mortality, and healthcare cost among non-communicable chronic diseases (2–4). Consequently, the burden of diabetes in 2021 included 37.8 million years of life lost and 41.4 million years lived with disability (3). Notably, T2DM frequently co-occurs with metabolic syndrome (MetS), present in roughly 85% of individuals (5, 6) and characterized by a cluster of abdominal obesity, elevated arterial blood pressure, atherogenic dyslipidemia, elevated plasma glucose, proinflammatory states, and prothrombotic states (7, 8). MetS is associated with a five-fold greater risk of T2DM, a two- to four-fold risk of stroke, and a three- to four-fold risk of myocardial infarction (9–11). This convergence of risk factors in subjects with both conditions markedly worsens prognosis. Therefore, addressing the co-morbidity of T2DM and MetS is a clinical imperative to mitigate their compounded risk and improve patient outcomes.

Traditional Chinese medicine (TCM) holds a unique position in managing diabetes and MetS, leveraging its holistic concept and multi-target regulatory approach (12, 13). TCM posits that health depends on the equilibrium of Yin, Yang, and the Five Elements (wood, fire, earth, metal, water), with Qi and blood mediating the interactions among these components. Imbalances triggered by cold, heat, emotional disturbances, or other factors lead to disease, and TCM treatment (e.g., herbal medicine) aims to restore balance and replenish Qi or blood (14). This theoretical framework has been increasingly supported by clinical evidence. A systematic review and meta-analysis of 26 randomized controlled trials (RCTs) concluded that TCM significantly improves glucose control and clinical indices in diabetic patients compared to placebo, thereby effectively delaying disease progression (12). Additionally, a systematic review of 25 RCTs demonstrated that TCM effectively manages multiple components of MetS. Benefits included reductions in body mass index (mean difference -1.16 [95% confidence interval -1.44, -0.89]), waist circumference (-2.64 [-3.42, -1.87]), triglycerides (-4.19 [-6.35, -2.03]), total cholesterol (-10.45 [-18.92, -1.98]), fasting blood glucose (-0.77 [-1.24, -0.29]), systolic blood pressure (-5.27 [-8.35, -2.19]), and increases in high-density lipoprotein (-3.60 [-6.73, -0.47]), with minimal adverse reactions (15). These findings position TCM as an evidence-based therapeutic option in the integrative management of T2DM and MetS.

The foundational principle guiding TCM practice is *Bian Zheng Lun Zhi* (syndrome differentiation followed by treatment procedures). This process represents a diagnostic synthesis of the pathological state of a disease, derived from a holistic analysis of an individual’s symptoms, signs, pulse, and tongue appearance (16). Current TCM syndrome definitions and diagnostic criteria rely largely on expert consensus, which may have inherent imprecision and variability (17). Additionally, it remains unclear whether TCM syndromes correspond to real-world entities or are primarily subjective notions (18). Recent research has found that syndrome differentiation exhibits correlations with individual variations in genomics, proteomics, and other omics (19, 20). Identifying specific biomarkers across these domains could therefore enhance the recognition and acceptance of TCM syndrome diagnosis (21). Despite these insights, the extent to which syndrome differentiation accurately capture discrete pathological states requires further validation. This study aims to identify prevalent TCM syndromes in individuals with T2DM and MetS with multivariate statistical methods and explore the potential associated factors. The findings could offer insights for exploring tailored therapeutic strategies for this comorbid population.

## Materials and methods

### Study settings

This cross-sectional study was conducted at Hong Kong Baptist University Mr. & Mrs. Chan Hon Yin Chinese Medicine Specialty Clinic and Good Clinical Practice Centre. Potential subjects were required to be domiciled in Hong Kong. Following an initial phone screening, eligibility was assessed by trained Chinese medicine practitioners (CMPs). Medical records were reviewed to confirm eligibility. The recruitment period spanned from November 2024 to December 2025. This study was approved by the Research Ethics Committee of HKBU (Ref no: REC/23-24/0564) and registered at ClinicalTrials.gov (Ref no: NCT06703684) (22). The reporting of this study followed the recommendations of the STROBE (Strengthening the Reporting of Observational Studies in Epidemiology) guideline (23).

### Study participants

Subjects who fulfilled all the following criteria were included: (1) Age between 18-75y. (2) Diagnosed with T2DM. (3) Diagnosed with metabolic syndrome according to Guideline for the prevention and treatment of T2DM in China (2020 edition) from Chinese Diabetes Society (24). People who meet 3 of the following diagnostic criteria or more can be diagnosed as MetS: a. Abdominal obesity: waist circumference ≥ 90cm for male and ≥ 85cm for female; b. Hyperglycemia: Fasting blood glucose ≥ 6.1mmol/L or 2-hour postprandial glucose ≥ 7.8mmol/L, and/or those who have been diagnosed with hyperglycemia and in treatment; c. Hypertension: blood pressure ≥ 130/85mmHg, and/or those who have been diagnosed with hypertension and in treatment; d. Fasting triglyceride ≥ 1.70mmol/L; e. Fasting high-density lipoprotein cholesterol < 1.04mmol/L. (4) Clearly understand the study and voluntarily sign the informed consent form.

Subjects who fulfilled any of the following criteria were excluded: (1) Diagnosed with type 1 diabetes, steroid-induced diabetes, gestational diabetes, or specific types of diabetes. (2) Diabetes accompanied by severe complications such as diabetic nephropathy, diabetic ketoacidosis, etc. (3) Secondary obesity (e.g., secondary to pituitary inflammation, tumor, etc.). (4) Secondary hypertension (e.g., pheochromocytoma, renal hypertension, etc.). (5) Secondary hyperlipidemia (e.g., hypothyroidism, nephrotic syndrome, etc.). (6) With severe heart, liver, or kidney disease or bleeding disorders, or with other serious diseases (e.g., cancer, dementia, etc.). (7) With severe mental disorders, speech or hearing impairments. (8) Pregnancy or lactation female. (9) Currently join other clinical trials.

### Study measurements

The primary outcome was TCM syndrome differentiation in patients with T2DM and MetS, as assessed by the Syndrome Differentiation Questionnaire for T2DM and MetS (SDQTM; **Supplementary Table 1**). The SDQTM is developed according to the Guideline for the Integrated Diagnosis and Treatment of Metabolic Syndrome (25), Guideline for the Integrated Diagnosis and Treatment of T2DM (26), and Guiding Principles for Clinical Research of New Chinese Medicine (27). It includes 69 items that capture disease nature (e.g., wind, yang deficiency) and location (e.g., heart, kidney) (28). Each item is a closed-ended question featuring a ranked scale with descriptors for symptom severity and frequency. The trained CMPs administered the SDQTM in a face-to-face consultation integrating the four TCM diagnostic methods.

Secondary outcomes included anthropometric indices, blood pressure, glycosylated hemoglobin (HbA1c), fasting glucose, lipid panel (total cholesterol, triglycerides, high- and low-density lipoprotein), sleep quality, physical activity, quality of life, and concurrent medications. Fat-to-muscle mass ratio (FMR) was defined as fat mass divided by muscle mass using a body composition analyzer (Mi Band 2, Xiaomi Corporation, Beijing, China) (29). The analyzer also provided basal metabolic rate (BMR). Waist-to-hip ratio (WHR) was calculated by dividing waist circumference, measured midway between the lowest ribs and the iliac crest (30), by hip circumference, measured at the widest portion of the buttocks. Blood pressure was measured in both arms initially, following which the arm with the higher systolic reading was selected for two additional measurements at 2-minute intervals with an automated upper-arm oscillometric device (CF155f, Rossmax International Ltd., Heerbrugg, Switzerland) after 15-minute rest, and the means were used for analysis (31). Following a 12-hour fast, finger capillary blood was collected to evaluate HbA1c (A1CNOW+, PTS Diagnostics, Indianapolis, USA), fasting glucose and lipid panel (CardioChek Plus, PTS Diagnostics, Indianapolis, USA).

Sleep quality was assessed using the Pittsburgh Sleep Quality Index (PSQI), a 19-item instrument whose items generate 7 component scores that are summed into a global score ranging from 0 to 21, with higher scores indicating poorer sleep quality (32). Fatigue was assessed on a 0-10 numerical rating scale, where 0 represented no fatigue and 10 represented the worst fatigue imaginable, with scores of 1-3, 4-6, and 7-10 indicating mild, moderate, and severe fatigue, respectively (33). Physical activity levels was assessed using International Physical Activity Questionnaire Short Form (IPAQ-SF), by recalling the time and days spent on 4 intensity levels (vigorous, moderate, walking, and sitting) over the previous 7 days (34). Dietary pattern and energy intake was assessed using a locally validated Food Frequency Questionnaire (FFQ) designed for older adults in Hong Kong (35). Audit of diabetes-dependent quality of Life (ADDQoL) assessed diabetes-specific quality of life through 2 overview items and 19 items covering domains include leisure, work, physical health, social relationships, and living conditions, with a lower mean weighted impact score indicating poorer diabetes-specific quality of life (36).

### Sample size calculation

The minimum required sample size was calculated using the single-population proportion formula (37), based on a T2DM with MetS prevalence of approximately 11.2% in Hong Kong (38). With a 95% confidence level, a precision of ±3%, and an assumed non-response rate of 10%, the study aimed to recruit 470 participants.

### Statistical analyses

Data analyses were performed using IBM Statistical Package Software for Social Science (SPSS) for macOS, version 29.0 (SPSS Inc., Chicago, IL, USA) and *R* statistical software (version 4.5.2) within the RStudio environment version 2025.09.2-418. Basic descriptive quantitative data were summarized as mean ± standard deviation (SD) if normally distributed, or as median and interquartile range (IQR) otherwise. Categorical data were expressed as numbers and percentages. Missing values were imputed by multiple imputation using the chained equation (MICE) method (39). The proportion of missing values for variables is summarized in **Supplementary Table 2**. For the comparison of quantitative data, one-way analysis of variance was used when the assumptions of normality and homogeneity of variance were met; otherwise, the Kruskal-Wallis test was employed. Comparisons of categorical data were conducted using the chi-square test or Fisher’s exact test, as appropriate. Univariate multinomial logistic regression was used to select variables with *P* < 0.05 for inclusion in the multiple multinomial logistic regression model. Variables of established clinical relevance were also included. Subsequently, a multinomial logistic regression analysis was performed to calculate adjusted odds ratios (ORs) and their 95% confidence intervals (CIs). A 2-sided value of *P* < 0.05 was considered statistically significant.

To ensure robust model estimation, symptoms with a prevalence of ≥30% were retained for analysis (40). Principal component analysis (PCA) was employed to reduce the dimensionality of TCM syndrome elements and elucidate their underlying structure. Prior to extraction, data suitability was assessed using Kaiser-Meyer-Olkin (KMO) measure and Bartlett’s test of sphericity. The number of components was determined based on eigenvalues (>1) and interpretability. Hierarchical cluster analysis was performed on these component scores using the between-groups average linkage method with Pearson correlation as the similarity measure. Inspection of the resulting dendrogram informed the initial estimation of the number of clusters. Based on this estimate, composite cluster scores were constructed by weighting the retained components according to their respective proportions of explained variance. The k-means algorithm was subsequently performed on these derived scores to obtain the final partition.

Latent class analysis (LCA) was conducted using the poLCA package in *R* statistical software (41). Models specifying between 3 to 6 latent classes were estimated and compared using Bayesian Information Criterion (BIC) and entropy. BIC is a parsimony measure that balances model complexity with goodness of fit, whereas entropy quantifies the degree of uncertainty in assigning individuals to classes (42). An entropy value approaching 1 indicates a high degree of certainty in class assignment, which is considered optimal. Each participant was subsequently assigned to the latent class corresponding to their highest Bayesian posterior probability. Finally, the adjusted Rand index (ARI) was computed to quantify the agreement between the classifications derived from LCA and the PCA-based cluster analysis (43).

### Funding declaration

This study was supported by the Chinese Medicine Development Fund, Government of the Hong Kong Special Administrative Region of China (23B2_067A_R1 to JL.Z), and the InnoHK initiative of the Innovation and Technology Commission of the Hong Kong Special Administrative Region Government (ITC RC/IHK/4/7 to ZX.B). The funder had no role in data collection, analysis, interpretation, manuscript writing, or the decision to submit.

## Results

### Participants characteristics

**Figure 1:**
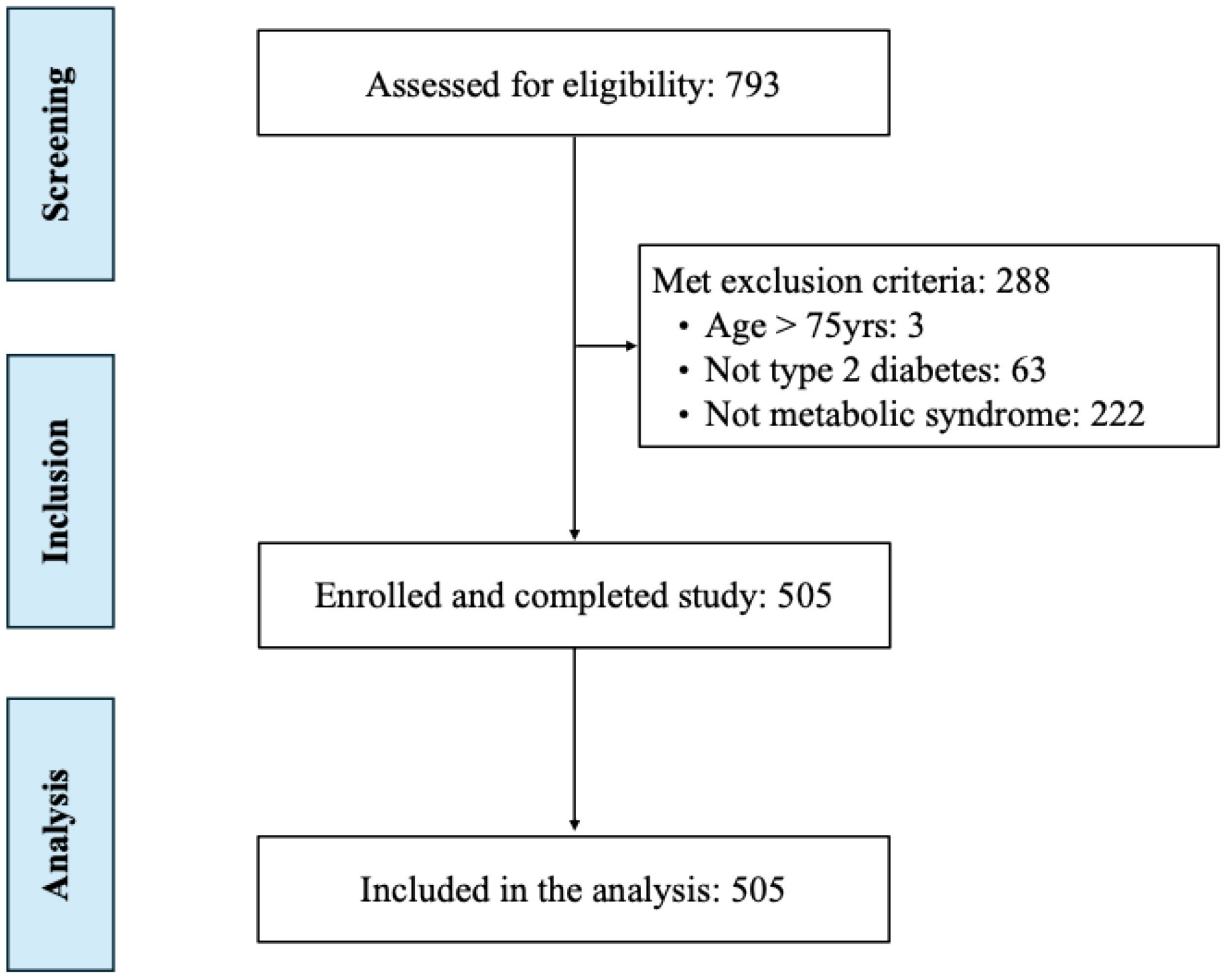
Flow diagram.

Of the 793 individuals screened, 505 were enrolled (**Figure 1**). The demographic and clinical characteristics are presented in **Tables 1** and **2**. The study population consisted entirely of Asian participants, predominantly aged 55 years or older (77.03%), 44.55% male, and classified as obese (73.27%). The median diabetes duration was 82.00 months, with a high prevalence of hypertension (88.12%) and hyperlipidemia (85.15%). Few participants reported current smoking (3.76%) or frequent alcohol consumption (4.55%). Regarding self-management, 49.91% monitored blood glucose at least weekly, 39.60% were physically inactive, and 21.78% reported adherence to a Dietary Approaches to Stop Hypertension (DASH)-style diet.

**Table 1:** Demographic and anthropometric characteristics of participants.

Characteristics	Total (n=505)
Age, years	61.00 (55.00, 66.00)
35–44	25 (4.95)
45–54	91(18.02)
55–64	231 (45.74)
65–75	158 (31.29)
Male gender, n (%)	225 (44.55)
Body mass index, kg/m^2^	27.15 (24.85, 29.77)
18.5–22.9	42 (8.32)
23.0–24.9	93 (18.42)
≥25.0	370 (73.27)
Educational attainment, n (%)	
Primary or below	27 (5.35)
Secondary	299 (59.21)
Post-secondary or above	179 (35.45)
Marital status, n (%)	
Married or living with partner	353 (69.90)
Single, separated, divorced, or widowed	152 (30.10)
Employment status, n (%)	
Full-time working	195 (38.61)
Part-time working	42 (8.32)
Retired/unemployed/housework	268 (53.07)
Occupation (n=228), n (%)	
Professional and associate professional	56 (23.63)
Skilled and semi-skilled worker	116 (48.95)
Unskilled worker	65 (27.43)
Household monthly income (HK$), n (%)	
<20,000	162 (32.08)
20,000–50,000	154 (30.50)
>50,000	67 (13.27)
Prefer not to answer	122 (24.16)
Living status, n (%)	
Living alone	67 (13.27)
Living with others	438 (86.73)
Home address, n (%)	
Hong Kong Island	78 (15.45)
Kowloon	168 (33.27)
New Territories	259 (51.29)
Diabetes duration, months	82.00 (34.00, 157.00)
Diabetes complications, n (%)	
Diabetic retinopathy	46 (9.11)
Diabetic neuropathy	6 (1.19)
Diabetic peripheral vascular disease	1 (0.20)
Antidiabetic medications, n (%)	
Oral medications	449 (88.91)
Injectable medications	59 (11.68)
Comorbidity, n (%)	
Hypertension	445 (88.12)
Hypertension duration, months	119.00 (58.50, 190.00)
Taking antihypertension agents	420 (94.38)
Hyperlipidemia	430 (85.15)
Hyperlipidemia duration, months	74.00 (36.00, 131.00)
Taking antihyperlipidemic agents	393 (91.40)
Cardiovascular diseases	66 (13.07)
Endocrine diseases	58 (11.49)
Digestive diseases	33 (6.53)
Respiratory diseases	27 (5.35)
Psychiatric diseases	29 (5.74)
Systolic blood pressure, mmHg	133.00 (124.30, 144.00)
Diastolic blood pressure, mmHg	84.00 (78.30, 90.00)
Heart rate, beats/minute	73.00 (64.70, 81.15)
Total cholesterol, mmol/L	3.95 (3.37, 4.63)
High-density lipoprotein, mmol/L	1.29 (1.09, 1.46)
Low-density lipoprotein, mmol/L	1.90 (1.41, 2.39)
Triglycerides, mmol/L	1.45 (1.11, 2.06)
Fasting glucose, mmol/L	7.20 (6.40, 8.30)
Glycated hemoglobin, %	6.20 (5.80, 6.60)
Waist-to-hip ratio	0.93 (0.89, 0.96)
Fat-to-muscle mass ratio	0.51 (0.36, 0.67)
Basal metabolic rate, kcal	1390.00 (1264.00, 1631.00)

Data are presented as mean ± SD, number (%), or median (IQR).

**Table 2:** Healthy behaviors and lifestyle characteristics of participants.

Characteristics	Total (n=505)
Smoking, n (%)	
Never	462 (91.49)
Former smoker	24 (4.75)
Current smoker	19 (3.76)
Alcohol consumption, n (%)	
Never	405 (80.20)
Occasional drinker (≤thrice/month)	77 (15.25)
Regular drinker (≥once/week)	23 (4.55)
Caffeine consumption, n (%)	
Almost none	117 (23.17)
1–2 cups/day	291 (57.62)
≥3 cups/day	97 (19.21)
Glucose monitor frequency	
Never	79 (15.64)
Only when unwell	13 (2.57)
1–2 times/quarter	82 (16.24)
1–2 times/month	71 (14.06)
1–2 times/week	154 (30.50)
≥3 times/week	98 (19.41)
Prefer not to answer	8 (1.58)
Fatigue score	5.00 (3.00, 6.00)
PSQI score	8.00 (5.00, 11.00)
Sleep duration, hour	6.00 (5.00, 7.00)
Energy expenditure (METs-min/week)	780.00 (252.00, 1810.50)
Time in vigorous PA, min	45.00 (20.00, 60.00)
Time in moderate PA, min	30.00 (20.00, 60.00)
Time in walking, min	45.00 (30.00, 60.00)
Physically inactive, n (%)	200 (39.60)
Physically active, n (%)	305 (60.40)
ADDQoL weighted score	-2.13 (-3.84, -1.11)
Leisure activities	-2.00 (-4.00, -1.00)
Working life	-2.00 (-4.00, 0.00)
Journeys	-2.00 (-4.00, 0.00)
Holidays	-2.00 (-4.00, -1.00)
Physical health	-2.00 (-4.00, -1.00)
Family life	-2.00 (-4.00, 0.00)
Friendship and social life	-2.00 (-4.00, 0.00)
Personal relationship	-2.00 (-4.00, 0.00)
Sex life	-2.00 (-4.00, 0.00)
Physical appearance	-2.00 (-4.00, 0.00)
Self-confidence	-2.00 (-4.00, 0.00)
Motivation	-2.00 (-4.00, -1.00)
People’s reaction	0.00 (-2.00, 0.00)
Feelings about future	-4.00 (-3.00, -1.00)
Financial situation	-2.00 (-4.00, 0.00)
Living conditions	0.00 (-2.00, 0.00)
Dependence on others	-1.00 (-3.00, 0.00)
Freedom to eat	-4.00 (-9.00, -2.00)
Freedom to drink	-4.00 (-6.00, -1.00)
DASH dietary pattern^a^, n (%)	110 (21.78)
Energy intake, kcal	1510.03 (1253.22, 1871.18)

Data are presented as mean ± SD, number (%), or median (IQR).

ADDQoL, Audit of Diabetes-Dependent Quality of Life; DASH, Dietary Approaches to Stop Hypertension; METs, metabolic equivalents; PA, physical activity; PSQI, Pittsburgh Sleep Quality Index.

^a^ Individuals meeting half of the DASH targets (DASH score ≥4.5) were considered DASH dietary pattern.

### Syndromes derived by PCA-based cluster analysis

**Figure 2:**
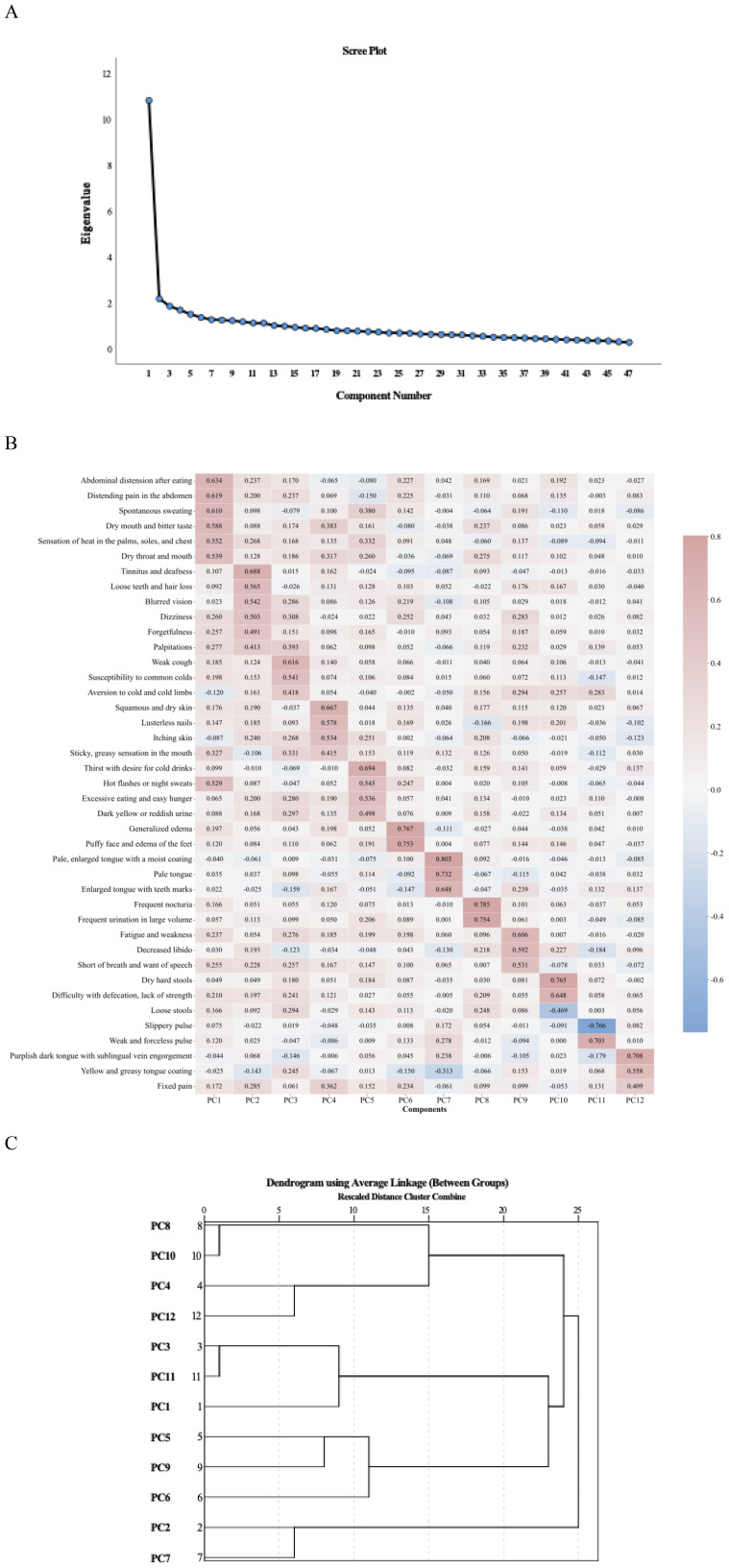
Factor extraction and syndrome clustering for T2DM with MetS. **(A)** The scree plot identified 12 components (eigenvalues >1). **(B)** The rotated component matrix yielded 12 components from 41 symptomatic items (loadings ≥0.4). **(C)** Cluster analysis of 12 components produced a dendrogram (cutoff distance of 15 defined 5 clusters).

From the SDQTM, 47 symptoms with occurrence frequencies >30% were included in subsequent analyses (**Supplementary Table 3**). PCA was applied to identify key symptomatic variables associated with T2DM and MetS. A 12-component model was selected as optimal for syndrome differentiation. The model showed good suitability for the analysis (KMO = 0.910; Bartlett’s test of sphericity: X^2^ = 7180.73, *P* < 0.001). Based on the scree plot criterion (eigenvalue >1), 12 components were retained (**Figure 2A**), accounting for 56.06% of the total variance. Following varimax rotation, 41 symptomatic items with loadings ≥0.4 were identified, constituting the 12 components (**Figure 2B**).

Hierarchical cluster analysis was applied to the scores of these 12 components, generating a dendrogram (**Figure 2C**). Guided by TCM theory and the cluster structure, a distance cutoff of 15 was chosen, yielding 5 distinct clusters. These corresponded to the following TCM syndrome patterns: Spleen and Kidney Yang Deficiency Syndrome (PC8, PC10), Qi and Yin Deficiency Syndrome (PC1, PC3, PC11), Kidney Essence Deficiency Syndrome (PC2, PC7), Yin and Yang Deficiency Syndrome (PC5, PC6, PC9), and Phlegm and Blood Stasis Syndrome (PC4, PC12). Based on composite cluster scores, participants were classified into these 5 syndromes, with the following distribution: Spleen and Kidney Yang Deficiency 17.62% (89/505), Qi and Yin Deficiency 22.97% (116/505), Kidney Essence Deficiency 10.89% (55/505), Yin and Yang Deficiency 22.97% (116/505), and Phlegm and Blood Stasis 25.54% (129/505).

### Syndromes derived by LCA

**Figure 3:**
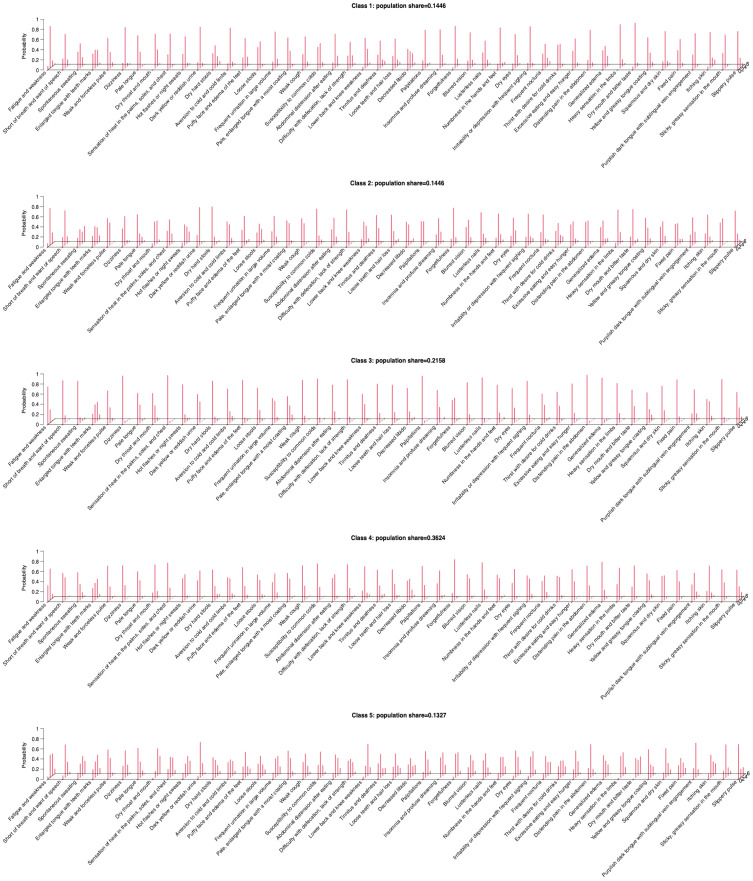
Estimation of the 5-class latent class model. Each group of red bars represents the conditional probabilities. Taller bars correspond to probabilities closer to 1 of a positive rating.

Latent class models with 3 to 6 classes were fitted, with model selection based on the BIC and entropy (**Supplementary Table 4**). To optimize interpretability and clinical utility, a 5-class solution was selected, which yielded an entropy of 0.948, indicating highly distinct and well-defined classes. The 5 latent classes, labeled according to their predominant symptom profiles (**Figure 3**; **Supplementary Table 5**), were as follows: (1) Class 1, Liver Depression and Spleen Deficiency Syndrome, was characterized by dry mouth and bitter taste, heavy sensation in the limbs, forgetfulness, fatigue and weakness, and irritability or depression with frequent sighing. (2) Class 2, Liver Depression and Spleen Deficiency with Qi and Yin Deficiency, with predominant symptoms included dark yellow or reddish urine, forgetfulness, fatigue and weakness, dry mouth and bitter taste, heavy sensation in the limbs, short of breath and want of speech, numbness in the hands and feet, and irritability or depression with frequent sighing. (3) Class 3, Liver and Kidney Yin Deficiency Syndrome, was characterized by forgetfulness, dry throat and mouth, dry mouth and bitter taste, itching skin, lower back and knee weakness, heavy sensation in the limbs, fatigue and weakness, and dry eyes. (4) Class 4, Qi and Yin Deficiency with Phlegm-Blood Stasis, was featured by dark yellow or reddish urine, purplish dark tongue with sublingual vein engorgement, distending pain in the abdomen, sticky, greasy sensation in the mouth, short of breath and want of speech, squamous and dry skin, dizziness, dry eyes, excessive eating and easy hunger, and palpitations. (5) Class 5, Phlegm and Blood Stasis Syndrome, was marked by purplish dark tongue with sublingual vein engorgement, forgetfulness, frequent urination in large volume, itching skin, and dark yellow or reddish urine. Based on the LCA, participants were allocated into the 5 classes with the following distribution: Class 1, 14.46% (73/505); Class 2, 14.46% (73/505); Class 3, 36.24% (183/505); Class 4, 13.27% (67/505); Class 5, 21.58% (109/505). The ARI between the LCA classification and the PCA-based clustering was 0.46, indicating a moderate level of agreement between the two classification methods.

### Potential associated factors of TCM syndromes

The multinomial logistic regression model for TCM syndromes derived from PCA-based cluster analysis was statistically significant (X^2^ = 353.209, *P* < 0.001, Nagelkerke *R^2^ =* 0.526). Using Phlegm and Blood Stasis Syndrome as the reference, significant factors of syndrome classification were gender, monthly household income, hypertension, fatigue severity, PSQI score, ADDQoL weighted score, and energy intake (**Table 3**). Significant syndrome-specific factors were identified: Spleen and Kidney Yang Deficiency Syndrome was associated with male gender (OR = 5.714, 95% CI = 1.490–21.908; *P* = 0.011), a household monthly income >HK$50,000 (OR = 3.469, 95% CI = 1.079–11.153; *P* = 0.037), the absence of hypertension (OR = 0.232, 95% CI = 0.069–0.781; *P* = 0.018), a lower basal metabolic rate (OR = 0.997, 95% CI = 0.993–1.000; *P* = 0.033), and a lower PSQI score (OR = 0.815, 95% CI = 0.738–0.900; *P* < 0.001). Qi and Yin Deficiency Syndrome was associated with male sex (OR = 17.614, 95% CI = 4.556–68.095; *P* < 0.001), a lower basal metabolic rate (OR = 0.997, 95% CI = 0.993–1.000; *P* = 0.033), a lower fatigue score (OR = 0.741, 95% CI = 0.632–0.868; *P* < 0.001), a lower PSQI score (OR = 0.770, 95% CI = 0.693–0.855; *P* < 0.001), and a higher ADDQoL weighted score (OR = 1.317, 95% CI = 1.095–1.585; *P* = 0.003). Kidney Essence Deficiency Syndrome was associated with a higher fatigue score (OR = 1.259, 95% CI = 1.006–1.576; *P* = 0.044), a higher PSQI score (OR = 1.203, 95% CI = 1.064–1.360; *P* = 0.003), and a higher energy intake (OR = 1.001, 95% CI = 1.000–1.002; *P* = 0.014). Yin and Yang Deficiency Syndrome was associated with male gender (OR = 9.394, 95% CI = 2.743–32.169; *P* < 0.001), a household monthly income <HK$20,000 (OR = 0.420, 95% CI = 0.200–0.882; *P* = 0.022) relative to the non-disclosure group, a lower fatigue score (OR = 0.825, 95% CI = 0.711–0.956; *P* = 0.011), and a lower PSQI score (OR = 0.877, 95% CI = 0.802–0.958; *P* = 0.004).

**Table 3:** Analysis of the factors influencing TCM syndromes derived by PCA-based cluster analysis (Phlegm and Blood Stasis Syndrome as reference group).

Variables	Spleen and Kidney Yang Deficiency Syndrome	Qi and Yin Deficiency Syndrome	Kidney Essence Deficiency Syndrome	Yin and Yang Deficiency Syndrome
Age, years	0.958 (0.916, 1.002)	0.985 (0.940, 1.031)	0.964 (0.916, 1.015)	0.981 (0.940, 1.024)
Gender				
Male	5.714 (1.490, 21.908)*	17.614 (4.556, 68.095)***	1.236 (0.285, 5.362)	9.394 (2.743, 32.169)***
Female	Ref	Ref	Ref	Ref
Body mass index, kg/m^2^	1.055 (0.966, 1.153)	0.989 (0.899, 1.087)	1.036 (0.934, 1.150)	0.963 (0.884, 1.050)
Household monthly income (HK$)				
<20,000	1.373 (0.578, 3.265)	0.843 (0.357, 1.991)	0.604 (0.229, 1.592)	0.420 (0.200, 0.882)*
20,000–50,000	1.563 (0.636, 3.842)	1.295 (0.549, 3.052)	0.612 (0.221, 1.696)	0.737 (0.350, 1.551)
>50,000	3.469 (1.079, 11.153)*	2.054 (0.657, 6.425)	0.910 (0.228, 3.626)	0.357 (0.109, 1.162)
Prefer not to answer	Ref	Ref	Ref	Ref
Without hypertension	0.232 (0.069, 0.781)*	1.243 (0.512, 3.020)	1.025 (0.297, 3.533)	0.619 (0.252, 1.520)
Without hyperlipidemia	1.309 (0.575, 2.976)	1.038 (0.436, 2.472)	0.247 (0.053, 1.157)	0.806 (0.347, 1.873)
Triglycerides, mmol/L	0.908 (0.640, 1.286)	1.103 (0.799, 1.524)	1.196 (0.753, 1.900)	0.838 (0.601, 1.168)
Glycated hemoglobin, %	1.195 (0.825, 1.732)	1.223 (0.830, 1.804)	1.074 (0.690, 1.672)	1.177 (0.819, 1.692)
Basal metabolic rate, kcal	0.997 (0.993, 1.000)*	0.997 (0.993, 1.000)*	0.999 (0.996, 1.002)	0.997 (0.994, 1.001)
Smoking				
Not current smoker	**/**	0.759 (0.130, 4.415)	1.519 (0.206, 11.217)	0.403 (0.089, 1.822)
Current smoker	Ref	Ref	Ref	Ref
Alcohol consumption				
Never	1.128 (0.228, 5.589)	2.036 (0.423, 9.806)	1.451 (0.241, 8.741)	1.326 (0.307, 5.730)
Occasional drinker (≤thrice/month)	1.311 (0.234, 7.346)	1.456 (0.263, 8.075)	1.380 (0.182, 10.480)	1.331 (0.273, 6.486)
Regular drinker (≥once/week)	Ref	Ref	Ref	Ref
Caffeine consumption, n (%)				
Almost none	1.954 (0.690, 5.533)	1.427 (0.527, 3.860)	0.469 (0.145, 1.513)	0.751 (0.303, 1.861)
1–2 cups/day	1.086 (0.437, 2.700)	0.897 (0.384, 2.094)	0.422 (0.160, 1.111)	0.482 (0.224, 1.037)
≥3 cups/day	Ref	Ref	Ref	Ref
Glucose monitor frequency^#^				
Rare	0.546 (0.219, 1.359)	0.564 (0.212, 1.498)	0.483 (0.120, 1.948)	0.723 (0.287, 1.820)
Occasional	0.540 (0.228, 1.281)	0.782 (0.322, 1.901)	2.206 (0.735, 6.621)	1.148 (0.497, 2.652)
Regular	0.521 (0.223, 1.219)	0.738 (0.305, 1.784)	1.436 (0.479, 4.308)	1.203 (0.531, 2.727)
Frequent	Ref	Ref	Ref	Ref
Fatigue score	0.937 (0.798, 1.099)	0.741 (0.632, 0.868)***	1.259 (1.006, 1.576)*	0.825 (0.711, 0.956)*
PSQI score	0.815 (0.738, 0.900)***	0.770 (0.693, 0.855)***	1.203 (1.064, 1.360)**	0.877 (0.802, 0.958)**
Energy expenditure				
Physically inactive	1.087 (0.579, 2.039)	0.821 (0.433, 1.558)	0.994 (0.445, 2.219)	0.802 (0.444, 1.446)
Physically active	Ref	Ref	Ref	Ref
ADDQoL weighted score	1.186 (0.998, 1.408)	1.317 (1.095, 1.585)**	0.918 (0.762, 1.105)	1.022 (0.877, 1.190)
Not DASH dietary pattern^a^	0.870 (0.406, 1.866)	0.940 (0.435, 2.030)	1.979 (0.604, 6.480)	0.833 (0.420, 1.654)
Energy intake, kcal	1.000 (0.999, 1.001)	1.001 (0.999, 1.000)	1.001 (1.000, 1.002)*	1.000 (0.999, 1.000)

Data are presented as adjusted OR (95% CI).

ADDQoL, Audit of Diabetes-Dependent Quality of Life; DASH, Dietary Approaches to Stop Hypertension; PSQI, Pittsburgh Sleep Quality Index; Ref, reference.

^#^ Glucose monitoring frequency was categorized as rare/none (never or only when unwell), occasional (1–2 times/quarter or 1–2 times/month), regular (1–2 times/week), and frequent (≥3 times/week).

^a^ Individuals meeting half of the DASH targets (DASH score ≥4.5) were considered DASH dietary pattern.

*P < 0.05, **P < 0.01, ***P < 0.001.Bold values indicate statistical significance.

The multinomial logistic regression model for latent classes derived from LCA was statistically significant (X^2^ = 335.673, *P* < 0.001, Nagelkerke *R^2^ =* 0.510). With Phlegm and Blood Stasis Syndrome as the reference, significant syndrome-specific factors were identified (**Table 4**): Liver Depression and Spleen Deficiency Syndrome was associated with older age (OR = 1.069, 95% CI = 1.010–1.130; *P* = 0.021), being female (OR = 0.040, 95% CI = 0.008–0.187; *P* < 0.001), a higher basal metabolic rate (OR = 1.005, 95% CI = 1.001–1.008; *P* = 0.014), never consuming alcohol compared to regular drinkers (OR = 0.141, 95% CI = 0.025–0.794; *P* = 0.026), a higher fatigue score (OR = 1.372, 95% CI = 1.139–1.652; *P* < 0.001), a higher PSQI score (OR = 1.235, 95% CI = 1.100–1.387; *P* < 0.001), and a lower ADDQoL weighted score (OR = 0.776, 95% CI = 0.628–0.958; *P* = 0.018). Liver Depression and Spleen Deficiency with Qi and Yin Deficiency was associated with female sex (OR = 0.053, 95% CI = 0.011–0.248; *P* < 0.001), a higher fatigue score (OR = 1.282, 95% CI = 1.074–1.530; *P* = 0.006), a higher PSQI score (OR = 1.225, 95% CI = 1.090–1.377; *P* < 0.001), and a lower ADDQoL weighted score (OR = 0.707, 95% CI = 0.575–0.869; *P* < 0.001). Liver and Kidney Yin Deficiency Syndrome was associated with lower triglyceride levels (OR = 0.732, 95% CI = 0.550–0.975; *P* = 0.033), a higher fatigue score (OR = 1.143, 95% CI = 1.002–1.303; *P* = 0.047), and a lower ADDQoL weighted score (OR = 0.803, 95% CI = 0.679–0.949; *P* = 0.010). Qi and Yin Deficiency with Phlegm-Blood Stasis was associated with female gender (OR = 0.121, 95% CI = 0.023–0.640; *P* = 0.013), minimal caffeine consumption (OR = 0.230, 95% CI = 0.065–0.817; *P* = 0.023), a higher fatigue score (OR = 1.545, 95% CI = 1.241–1.924; *P* < 0.001), a higher PSQI score (OR = 1.564, 95% CI = 1.362–1.796; *P* < 0.001), and a lower ADDQoL weighted score (OR = 0.681, 95% CI = 0.546–0.850; *P* < 0.001).

**Table 4:** Analysis of the factors influencing TCM syndromes derived by LCA (Class 5, Phlegm and Blood Stasis Syndrome as reference group).

Variables	Liver Depression and Spleen Deficiency Syndrome	Liver Depression and Spleen Deficiency with Qi and Yin Deficiency	Liver and Kidney Yin Deficiency Syndrome	Qi and Yin Deficiency with Phlegm-Blood Stasis
Age, years	1.069 (1.010, 1.130)*	0.983 (0.934, 1.034)	1.000 (0.960, 1.042)	0.988 (0.935, 1.044)
Gender				
Male	0.040 (0.008, 0.187)***	0.053 (0.011, 0.248)***	0.304 (0.091, 1.013)	0.121 (0.023, 0.640*
Female	Ref	Ref	Ref	Ref
Body mass index, kg/m^2^	0.934 (0.833, 1.047)	1.101 (0.991, 1.224)	1.026 (0.942, 1.119)	1.110 (0.985, 1.252)
Household monthly income (HK$)				
<20,000	1.127 (0.442, 2.870)	0.911 (0.341, 2.433)	0.713 (0.333, 1.527)	0.821 (0.279, 2.412)
20,000–50,000	0.914 (0.344, 2.427)	0.759 (0.284, 2.027)	1.049 (0.504, 2.187)	0.557 (0.184, 1.682)
>50,000	0.269 (0.063, 1.144)	0.722 (0.211, 2.470)	0.701 (0.285, 1.724)	0.375 (0.088, 1.587)
Prefer not to answer	Ref	Ref	Ref	Ref
Without hypertension	1.295 (0.482, 3.480)	0.412 (0.129, 1.314)	0.505 (0.224, 1.139)	0.974 (0.280, 3.393)
Without hyperlipidemia	0.871 (0.321, 2.369)	0.730 (0.263, 2.028)	1.016 (0.486, 2.123)	0.659 (0.190, 2.283)
Triglycerides, mmol/L	0.776 (0.529, 1.138)	1.038 (0.712, 1.514)	0.732 (0.550, 0.975)*	1.281 (0.822, 1.995)
Glycated hemoglobin, %	0.860 (0.537, 1.378)	1.001 (0.651, 1.539)	0.972 (0.689, 1.371)	0.995 (0.625, 1.583)
Basal metabolic rate, kcal	1.005 (1.001, 1.008)*	1.003 (0.999, 1.006)	1.001 (0.998, 1.004)	1.002 (0.998, 1.006)
Smoking				
Not current smoker	0.995 (0.130, 7.595)	0.550 (0.101, 2.992)	1.584 (0.360, 6.968)	2.131 (0.244, 18.617)
Current smoker	Ref	Ref	Ref	Ref
Alcohol consumption				
Never	0.141 (0.025, 0.794)*	0.997 (0.085, 11.724)	0.299 (0.075, 1.194)	0.538 (0.070, 4.148)
Occasional drinker (≤thrice/month)	0.309 (0.048, 2.010)	1.002 (0.074, 13.512)	0.581 (0.131, 2.574)	0.471 (0.048, 4.646)
Regular drinker (≥once/week)	Ref	Ref	Ref	Ref
Caffeine consumption, n (%)				
Almost none	0.589 (0.187, 1.857)	0.416 (0.136, 1.273)	0.675 (0.291, 1.569)	0.230 (0.065, 0.817)*
1–2 cups/day	0.987 (0.368, 2.644)	0.477 (0.186, 1.227)	0.688 (0.337, 1.407)	0.409 (0.145, 1.153)
≥3 cups/day	Ref	Ref	Ref	Ref
Glucose monitor frequency^#^				
Rare	1.692 (0.547, 5.228)	0.603 (0.204, 1.784)	0.939 (0.397, 2.223)	0.515 (0.135, 1.960)
Occasional	1.813 (0.638, 5.150)	0.562 (0.202, 1.565)	1.173 (0.541, 2.547)	1.297 (0.415, 4.057)
Regular	1.302 (0.462, 3.674)	0.687 (0.257, 1.835)	0.936 (0.439, 1.996)	0.911 (0.295, 2.818)
Frequent	Ref	Ref	Ref	Ref
Fatigue score	1.372 (1.139, 1.652)***	1.282 (1.074, 1.530)**	1.143 (1.002, 1.303)*	1.545 (1.241, 1.924)***
PSQI score	1.235 (1.100, 1.387)***	1.225 (1.090, 1.377)***	1.079 (0.982, 1.185)	1.564 (1.362, 1.796)***
Energy expenditure				
Physically inactive	0.792 (0.383, 1.635)	1.334 (0.647, 2.751)	0.844 (0.485, 1.471)	1.032 (0.444, 2.395)
Physically active	Ref	Ref	Ref	Ref
ADDQoL weighted score	0.776 (0.628, 0.958)*	0.707 (0.575, 0.869)***	0.803 (0.679, 0.949)*	0.681 (0.546, 0.850)***
Not DASH dietary pattern^a^	0.885 (0.383, 2.046)	2.445 (0.918, 6.513)	0.893 (0.462, 1.727)	2.997 (0.885, 10.148)
Energy intake, kcal	1.000 (0.999, 1.001)	1.000 (0.999, 1.001)	1.000 (0.999, 1.000)	1.001 (0.999, 1.001)

Data are presented as adjusted OR (95% CI).

ADDQoL, Audit of Diabetes-Dependent Quality of Life; DASH, Dietary Approaches to Stop Hypertension; PSQI, Pittsburgh Sleep Quality Index; Ref, reference.

^#^ Glucose monitoring frequency was categorized as rare/none (never or only when unwell), occasional (1–2 times/quarter or 1–2 times/month), regular (1–2 times/week), and frequent (≥3 times/week).

^a^ Individuals meeting half of the DASH targets (DASH score ≥4.5) were considered DASH dietary pattern.

*P < 0.05, **P < 0.01, ***P < 0.001.Bold values indicate statistical significance.

## Discussion

### Data-driven classification of TCM syndromes in comorbid T2DM and MetS

Syndrome differentiation is fundamental to TCM diagnosis and treatment. Although grounded in expert consensus, its application in clinical practice is complicated by significant population heterogeneity and complex disease phenotypes (44, 45). To address this, this cross-sectional study represents the first investigation in Hong Kong to establish data-driven support for TCM syndrome differentiation in T2DM and MetS, utilizing multivariate techniques applied to clinical symptoms to promote objective diagnostic strategies. The application of PCA-based analysis and LCA generated distinct yet complementary classifications. PCA-derived analysis identified 5 symptom-based syndromes, and subsequent regression modeling, using Phlegm and Blood Stasis Syndrome as the reference, established a differential diagnostic framework that delineated specific male-predominant deficiency subtypes characterized by contextual socioeconomic and physiological factors. Conversely, LCA identified 5 latent subgroups, defining a female-predominant spectrum of integrated phenotypes marked by significant functional impairment and specific biomarker correlations. Consequently, PCA provides a symptom-oriented taxonomic framework, while LCA delineates person-centered phenotypic profiles, together furnishing a multidimensional perspective on syndrome heterogeneity in T2DM with MetS.

The agreement between the LCA classification and PCA-based clustering was moderate, as quantified by an ARI of 0.46. This indicates a partial but meaningful overlap in how the two data-driven methods conceptualize the syndromic structure of the population. Beyond this structural concordance, a key substantive convergence was the identification of Phlegm and Blood Stasis Syndrome as a prevalent and central entity by both analytical paradigms. This finding aligns with prior TCM research in T2DM (46). Epidemiological studies have consistently identified blood stasis and phlegm-dampness as common patterns in T2DM, with a study reporting blood stasis in collaterals as a primary syndrome in 9.41% of patients (47). Furthermore, large-scale text mining analyses of TCM literature have systematically categorized blood stasis and phlegm-dampness among the core excess syndromes for T2DM (48), and classical TCM theories on metabolic disorders also emphasize the central role of phlegm-turbidity and the need for simultaneous treatment of phlegm and blood stasis (49). The prominence of this syndrome in our study is biologically plausible, as its conceptual framework is closely consistent with established pathophysiological hallmarks of T2DM and MetS, including chronic inflammation, insulin resistance, and microvascular complications (50). This consensus across divergent methodological approaches reinforces the robustness of Phlegm and Blood Stasis as a core pathological construct in this comorbid condition.

Beyond the point of convergence, the discrepancies between the syndromic constructs identified by PCA and LCA stem directly from their divergent methodological architectures. PCA adopts a variable-oriented framework, reducing data dimensionality by modeling covariance structures among symptoms to extract latent dimensions. Consequently, the syndromes derived from PCA, such as Spleen-Kidney Yang Deficiency, are operationally defined as latent statistical factors or idealized symptom combinations that serve as differential labels within a taxonomy based on variable correlation. In contrast, LCA is a person-oriented, finite mixture modeling technique that explains population heterogeneity by identifying discrete latent subgroups and assigning individuals to mutually exclusive categories based on distinct probabilistic profiles of indicators (51, 52). Therefore, the subgroups identified by LCA, such as Liver Depression and Spleen Deficiency, are operationally defined as probabilistic latent categories of individuals, representing cohesive phenotypic clusters characterized by a holistic set of symptoms. This foundational conceptual dichotomy between PCA’s analysis of symptom covariance and LCA’s modeling of population heterogeneity preconstructs the nature of their respective syndromic outputs.

### Syndrome-associated factors in comorbid T2DM and MetS

The multinomial logistic regression revealed distinct determinants for the syndromes derived from PCA and LCA. PCA-based syndromes were associated with a profile of differential contextual and physiological correlates, including specific socioeconomic linkages and features such as lower basal metabolic rate. In contrast, the LCA-identified subgroups were associated with a profile of integrated clinical severity. This profile was characterized by significant functional impairment, evidenced by elevated fatigue and sleep disturbance scores, diminished quality of life, and specific biomarkers such as lower triglyceride levels. A key point of convergence between the PCA-derived Kidney Essence Deficiency Syndrome and the LCA-identified deficiency syndromes is the strong association with the clinical burdens of fatigue and poor sleep quality. Within T2DM and MetS, these symptoms are recognized as core clinical features, with significant fatigue affecting an estimated 50-65% of patients (53, 54), and sleep disturbances impacting up to 77% (55). These conditions form a bidirectional vicious cycle with metabolic dysregulation, accelerating disease progression and elevating complication risk, which underscores their integral role in clinical management and the need for further mechanistic investigation.

The LCA-derived Liver and Kidney Yin Deficiency syndrome demonstrated a unique association with lower triglyceride levels, a correlation absent in other subgroups. This finding warrants interpretation through pharmacological context of the cohort, where long-term therapy likely lowered and compressed the triglyceride range, accentuating the specificity of this association. This correlation was independent of basal metabolic rate and energy intake, indicating it is not a proxy for overall energy deficit. In TCM theory, the syndrome pathogenesis of Yin deficiency with internal heat and depletion of essential substances may correlate with a specific metabolic phenotype. This phenotype could involve altered lipid synthesis or mobilization, manifesting as a tendency toward even lower triglyceride levels under comparable treatment. Therefore, this association may reveal a distinct, syndrome-specific metabolic pattern discernible within a pharmacologically managed population.

### Implications for TCM syndrome research and therapeutic strategies

This study provides a methodological investigation for advancing TCM syndrome research through complementary data-driven approaches. It applies two distinct approaches, symptom-oriented PCA from person-centered LCA, to capture clinical heterogeneity in T2DM with MetS (56). The framework grounds theoretical syndromes in empirical patient clusters, reflecting real-world complexity. These empirically derived subtypes may help explore pathophysiological mechanisms, potentially facilitating a transition from descriptive classification toward mechanism-driven investigation. Furthermore, cross-methodological validation of core entities like Phlegm and Blood Stasis supports prioritizing consensus-validated syndromes in future investigations.

The data-driven classifications offer insights for personalizing T2DM and MetS management. They identify a subgroup characterized by profound functional impairment and distinct biomarker patterns, necessitating integrated interventions that address both metabolic control and core symptoms like fatigue. Furthermore, distinct syndrome correlates allow stratification of prevention and treatment based on symptom patterns and underlying risk profiles. This supports syndrome-specific recommendations, such as tailored lifestyle, nutritional, or herbal regimens. Realizing this potential requires longitudinal studies to examine whether these subtypes predict differential responses to tailored interventions, thereby advancing a personalized integrative medicine model.

### Strengths and limitations

This study offers new insights for the research on TCM syndrome differentiation in T2DM with MetS. Methodologically, prior research has relied on a single methodological approach, either for syndrome discovery (e.g., LCA or latent tree analysis) or for predictive modeling (e.g., using supervised machine learning) (57–60). In this context, the primary strength of the present study lies in its comparative design, applying both PCA and LCA to the same cohort using a common set of clinical symptoms for syndrome differentiation, which allows for a clear dissection of how analytical philosophy influences syndromic classification. The use of a common reference group in regression models further enhanced the comparability of the associated factors. However, several limitations must be acknowledged. The cross-sectional design precludes causal inference regarding the identified associations. Furthermore, the sample was drawn from a specific clinical setting, which may limit the generalizability of the prevalence of derived syndromes to other populations. Additionally, participants with severe complications (e.g., diabetic nephropathy, ketoacidosis) or secondary metabolic disorders were excluded, thereby restricting applicability of the findings to these advanced disease stages. Moreover, multinomial logistic regression revealed that TCM syndrome classification was associated with gender and income, and future studies with larger samples are needed to clarify potential interactions. PCA is intended for continuous variables, yet questionnaire data are frequently ordinal, and future studies may therefore explore alternative approaches to ascertain whether comparable results can be obtained. Finally, the syndrome patterns, while clinically interpretable, require validation in independent longitudinal cohorts to confirm the stability.

## Conclusion

This study demonstrates the utility of applying PCA and LCA to delineate TCM syndromes in comorbid T2DM and MetS, yielding complementary classifications. PCA derived 5 symptom-based syndromes: Spleen and Kidney Yang Deficiency Syndrome, Qi and Yin Deficiency Syndrome, Kidney Essence Deficiency Syndrome, Yin and Yang Deficiency Syndrome, and Phlegm and Blood Stasis Syndrome. LCA identified 5 patient-centered subtypes: Liver Depression and Spleen Deficiency Syndrome, Liver Depression and Spleen Deficiency with Qi and Yin Deficiency, Liver and Kidney Yin Deficiency Syndrome, Qi and Yin Deficiency with Phlegm-Blood Stasis, and Phlegm and Blood Stasis Syndrome. Key substantive findings include the cross-methodological validation of Phlegm and Blood Stasis as a core syndrome, the strong association of both PCA-derived Kidney Essence Deficiency Syndrome and the LCA-identified deficiency syndromes with the clinical burdens of fatigue and poor sleep quality, and the unique association of the LCA-derived Liver and Kidney Yin Deficiency subtype with lower triglyceride levels. Given the cross-sectional design, this work provides a preliminary empirical basis for future longitudinal studies that may explore whether the derived subtypes differ in treatment response or long-term prognosis, with further validation required before clinical relevance can be established.

## Data Availability

Individual, de-identified patient data are available at the request of investigators who propose to use the data with a methodologically sound research proposal.
