# Supplementary material for: Traditional Chinese medicine syndrome patterns and associated factors in adults with type 2 diabetes and metabolic syndrome: a data-driven analysis
Source: Front Endocrinol (Lausanne). 2026 Jun 17;17:1843491. doi: 10.3389/fendo.2026.1843491 (PMC13318567; doi:10.3389/fendo.2026.1843491)
Supplement: Supplementary file 1 [file DataSheet1.docx]

**Supplementary appendix**

This appendix formed part of the original submission.

Supplement to: Jialing Zhang, Zhilin Lin, Shuyan Zhong, et al. Traditional Chinese medicine syndrome patterns and associated factors in adults with type 2 diabetes and metabolic syndrome: a data-driven analysis.

| **Table 1.** The Syndrome Differentiation Questionnaire for T2DM and MetS (SDQTM) | |
| --- | --- |
| 1. Fatigue and weakness | - None - Mild (Persistent decrease in energy and endurance, persists despite rest, but does not interfere with daily activities) - Moderate (Persistent mental fatigue, feeling weak with routine activities) - Severe (Persistent mental exhaustion, feeling weak even at rest) |
| 2. Short of breath and want of speech | - None - Mild (Shortness of breath after routine activities, tendency towards disinclination to speak, does not interfere with work or daily life) - Moderate (Occasional shortness of breath even at rest, disinclination to speak) - Severe (Persistent shortness of breath at rest, weak voice, fatigued speech, interfere with work or daily life) |
| 3. Spontaneous sweating | - None - Mild (Slight sweating at rest during the day, unrelated to eating or hot weather; increased with activity) - Moderate (Noticeable sweating at rest during the day, unrelated to eating or hot weather; profuse sweating with activity) - Severe (Profuse sweating at rest during the day, unrelated to eating or hot weather; excessive sweating with activity) |
| 4. Pale or sallow yellow complexion | - None - Mild (Pale complexion, lacklustre) - Moderate (Pale complexion, bloodless or sallow) - Severe (Ghastly pale, bloodless complexion, or sallow with mild oedema) |
| 5. Pale lips and fingernails | - None - Mild (Dry lips and nails, lacking redness) - Moderate (Dry lips and nails, pale and lacklustre) - Severe (Dry lips and nails, ghastly pale and lacklustre) |
| 6. Dizziness | - None - Mild (Occasional, unrelated to postural change) - Moderate (Frequent, unrelated to postural change) - Severe (Recurrent, unrelated to postural change, difficult to alleviate) |
| 7. Dry throat and mouth | - None - Mild (Frequent mild throat dryness during the day, relieved by drinking a little water) - Moderate (Persistent throat dryness day or night, relieved by drinking water) - Severe (Unbearable throat dryness day or night, not relieved by drinking water) |
| 8. Sensation of heat in the palms, soles, and chest | - None - Mild (Hot palms and soles, occasional restlessness for no reason) - Moderate (Hot palms and soles, desire for extremity exposure, frequent restlessness for no reason) - Severe (Extremely hot palms and soles, craving for cold objects, persistent restlessness for no reason) |
| 9. Hot flashes or night sweats | - None - Mild (Mild heat intolerance, mild sweating, occasional night sweats) - Moderate (Marked heat intolerance, occasional sweating, frequent night sweats) - Severe (Pronounced heat intolerance, persistent sweating, nightly drenching sweats) |
| 10. Aversion to cold and cold limbs | - None - Mild (Occasional coldness of the extremities, not requiring additional clothing, typically triggered by wind exposure) - Moderate (Frequent coldness of the extremities, more pronounced than in others, often occurring at night) - Severe (Marked generalised coldness, necessitating clothing appropriate for a warmer season than peers) |
| 11. Puffy face and oedema of the feet | - None - Mild (Facial oedema upon awakening) - Moderate (Persistent bilateral lower limb oedema with pitting) - Severe (Severe periorbital and facial oedema lasting throughout the day; lower limb oedema with deep and persistent pitting, often accompanied by generalised oedema) |
| 12. Weak cough | - None - Mild (Mildly diminished cough sound) - Moderate (Notably weak cough) - Severe (Markedly diminished and weak cough) |
| 13. Susceptibility to common colds | - None - Mild (More than 6 colds per year) - Moderate (More than 10 colds per year) - Severe (More than 12 colds per year) |
| 14. Reduced food intake and poor appetite | - None - Mild (Lack of appetite, food intake unchanged) - Moderate (Lack of appetite, food intake reduced by 1/3) - Severe (Food intake reduced by over 2/3) |
| 15. Abdominal distension after eating | - None - Mild (Mild abdominal distension, resolving within 30 minutes, does not interfere with daily life, no symptomatic medication required) - Moderate (Significant abdominal distension persisting for 30 minutes to 1 hour, partially interfere with daily activities or requiring symptomatic medication) - Severe (Marked abdominal distension persisting beyond 2 hours, significantly interfere with daily functioning, or unresponsive to symptomatic medication) |
| 16. Lower back and knee weakness | - None - Mild (Intermittent lumbar soreness and knee weakness without trauma or major exertion, occasional pain) - Moderate (Persistent lumbar soreness, knee weakness, and lower limb heaviness without trauma or major exertion) - Severe (Intense lumbar pain, pronounced knee weakness with reluctance to ambulate; requiring pharmacological intervention, all independent of trauma or major exertion) |
| 17. Tinnitus and deafness | - None - Mild (Intermittent tinnitus, occasional episodes lasting seconds, no hearing impairment) - Moderate (Frequent tinnitus, episodes last minutes, mild hearing impairment) - Severe (Persistent tinnitus, described as high-pitched cicada-like or low-pitched train-like sounds, moderate hearing impairment) |
| 18. Loose teeth and hair loss | - None - Mild (Mild dental looseness or minimal hair loss) - Moderate (Noticeable dental looseness or substantial hair loss) - Severe (Tooth loss, dental and hair sparsity, or severe hair loss) |
| 19. Decreased libido | - None - Mild (Decreased libido) - Moderate (Occasional sexual desire) - Severe (Erectile dysfunction or infertility) |
| 20. Palpitations | - None - Mild (Intermittent palpitations unrelated to hunger, noticeable after physical exertion) - Moderate (Frequent palpitations unrelated to hunger, exacerbated by minimal physical activity) - Severe (Persistent palpitations unrelated to hunger, accompanied by heightened anxiety, occurring even at rest, requiring pharmacological control) |
| 21. Insomnia and profuse dreaming | - None - Mild (Frequent nocturnal awakenings, non-restorative or early-morning awakening without identifiable cause, occupational functioning unimpaired) - Moderate (Sleep duration <4 hours/night without apparent cause, fragmented sleep with excessive dreaming, able to maintain work) - Severe (Persistent insomnia without identifiable cause, unable to maintain normal occupational functioning) |
| 22. Forgetfulness | - None - Mild (Frequent recent memory lapses without impairment of occupational or daily functioning) - Moderate (Frequent recent memory lapses with significant impairment of occupational or daily functioning) - Severe (Impairment of both recent and remote memory, compromising independent living) |
| 23. Blurred vision | - None - Mild (Occasional floaters without visual blurring or distortion) - Moderate (Multiple floaters or mild visual blurring/distortion) - Severe (Large dark patches or severe visual blurring/distortion) |
| 24. Lustreless nails | - None - Mild (Nail surface lacks lustre and smoothness) - Moderate (Nail thinning and brittleness with cracking) - Severe (Marked nail dystrophy with dryness and atrophy) |
| 25. Numbness in the hands and feet | - None - Mild (Occasional episodes unrelated to external compression) - Moderate (Frequent episodes unrelated to external compression, amenable to relief) - Severe (Frequent episodes unrelated to external compression, refractory to relief) |
| 26. Dry eyes | - None - Mild (Frequent ocular dryness and visual fatigue, unrelated to contact lens use or prolonged visual activity) - Moderate (Recurrent ocular dryness and discomfort, with intermittent blurred vision, unrelated to contact lens use or prolonged visual activity) - Severe (Marked ocular dryness with persistent visual dimness, unrelated to contact lens use or prolonged visual activity) |
| 27. Irritability or depression with frequent sighing | - None - Mild (Occasional episodes without identifiable cause) - Moderate (Frequent episodes without identifiable cause) - Severe (Highly susceptible to episodes without identifiable cause, difficult to self-control) |
| 28. Cold pain in the abdomen | - None - Mild (Abdominal chilliness with occasional mild discomfort) - Moderate (Abdomen hypothermia with episodic dull pain, duration ≤2 hours) - Severe (Marked abdomen cold sensation with pronounced and persistent pain, duration >2 hours or continuous) |
| 29. Thirst with desire for cold drinks | - None - Mild (Slightly increased fluid intake with preference for cool beverages) - Moderate (Fluid intake elevated by ≥50% relative to baseline, with preference for cold beverages) - Severe (Fluid intake elevated by ≥100% relative to baseline, with strong craving for cold beverages) |
| 30. Excessive eating and easy hunger | - None - Mild (Slightly increased appetite, with food intake increased by <50% relative to baseline) - Moderate (Hunger occurring within 2 hours after meals, with food intake increased by 50-100% relative to baseline) - Severe (Persistent hunger throughout the day, with food intake increased by >100% relative to baseline) |
| 31. Fullness in chest and hypochondrium | - None - Mild (Slight fullness in the chest and hypochondrium, spontaneously resolving) - Moderate (Pronounced fullness in the chest and hypochondrium, partially interfering with daily activities) - Severe (Marked fullness in the chest and hypochondrium, significantly impairing work and rest, necessitating symptomatic treatment) |
| 32. Distending pain in the abdomen | - None - Mild (Mild epigastric and abdominal distension, aggravated postprandially, intermittent) - Moderate (Notable epigastric and abdominal distension, alleviated by fasting or passing flatus, accompanied by reduced food intake) - Severe (Marked epigastrium and abdominal rigidity and distension, persistent throughout the day; in severe cases, accompanied by abdominal pain and tenderness) |
| 33. Generalized oedema | - None - Mild (Persistent periorbital oedema upon awakening) - Moderate (Persistent periorbital and bilateral lower extremity oedema) - Severe (Generalised, systemic oedema) |
| 34. Heavy sensation in the limbs | - None - Mild (Slight heaviness in the limbs, with fatigue developing after physical exertion) - Moderate (Marked heaviness in the limbs, with fatigue even at rest) - Severe (Generalised sensation of heaviness throughout the body, with profound, unrelenting fatigue) |
| 35. Dry mouth and bitter taste | - None - Mild (Nocturnal oral dryness with bitter taste upon awakening) - Moderate (Persistent dry mouth with diminished salivation and bitter taste impairing food perception) - Severe (Marked dry mouth with desire to drink, accompanied by bitter and astringent taste) |
| 36. Dusky or dull dark complexion | - None - Mild (Facial skin displays a mildly dusky or dull complexion) - Moderate (Facial skin displays a pronounced dusky or dull complexion) - Severe (Facial skin displays a deeply dusky or markedly dark, dull complexion) |
| 37. Purplish dark lips | - None - Mild (Purplish-dark discolouration of the lips without ecchymoses) - Moderate (Purplish-dark discolouration of the lips and tongue, with multiple ecchymoses and petechiae) - Severe (Deep purplish-dark discolouration of the lips and tongue, with extensive ecchymoses) |
| 38. Squamous and dry skin | - None - Mild (Localised roughness and dryness of the skin) - Moderate (Diffuse rough and dry skin with hyperkeratosis and scaling, underlying erythema may form coalesce into patches) - Severe (Extensive rough, dry, and hyperkeratotic skin, resembling ichthyosis or snake-like skin) |
| 39. Fixed pain | - None - Mild (Occasional localised stabbing pain in a specific region) - Moderate (Intermittent stabbing pain involving different regions) - Severe (Persistent stabbing pain affecting multiple regions) |
| 40. Petechiae on the skin | - None - Mild (Petechiae or ecchymosis in ≤2 sites, attributable to trauma) - Moderate (Petechiae or ecchymosis in ≥3 sites) - Severe (Petechiae, ecchymosis, or cyanosis in ≥3 sites, occurring spontaneously) |
| 41. Nausea | - None - Mild (Nausea occurring 1-2 times per day) - Moderate (Nausea occurring 3-4 times per day) - Severe (Nausea occurring >4 times per day or nearly constantly) |
| 42. Vomiting | - None - Mild (Vomiting 1-2 times per day) - Moderate (Vomiting 3-4 times per day) - Severe (Vomiting >4 times per day or recurrent/protracted vomiting) |
| 43. Itching skin | - None - Mild (Occasional localised pruritus) - Moderate (Intermittent pruritus affecting different skin regions) - Severe (Persistent or widespread pruritus involving multiple skin regions; intolerable) |
| 44. Sticky, greasy sensation in the mouth | - None - Mild (Mild oral stickiness without impact on appetite) - Moderate (Mild oral stickiness with reduced appetite) - Severe (Marked oral stickiness causing significant discomfort and aversion to food and beverage) |
| 45. Dark yellow or reddish urine | - None - Mild (Yellow urine with a sensation of heat, without affecting urination) - Moderate (Dark yellow urine with burning sensation, impaired urination) - Severe (Yellow-red urine with burning sensation, dysuria and pain on urination) |
| 46. Frequent urination in large volume | - None - Mild (Increased urinary frequency and volume, ≤10 times daily) - Moderate (Increased urinary frequency and volume, 10-15 times daily) - Severe (Markedly increased urinary volume, >15 times daily) |
| 47. Frequent nocturia | - None - Mild (Nocturia 2 times per night) - Moderate (Nocturia 3 times per night) - Severe (Nocturia 4 or more times per night) |
| 48. Scanty urine | - None - Mild (Slightly decreased urine output) - Moderate (Urine output reduced to 1/2 of baseline) - Severe (Urine output reduced to less than 1/3 of baseline) |
| 49. Dry hard stools | - None - Mild (Dry and hard stools, bowel movement daily) - Moderate (Constipated stools, bowel movement once every two days) - Severe (Severely constipated stools, bowel movement once every several days) |
| 50. Loose stools | - None - Mild (Loose and unformed stools, once daily) - Moderate (Loose and soft stools, 2-3 times daily) - Severe (Loose and soft stools, more than 3 times daily) |
| 51. Difficulty with defecation, lack of strength | - None - Mild (Straining required for defecation) - Moderate (Significant effort needed to evacuate stool) - Severe (Defecation difficult despite repeated straining; requires manual assistance or pharmacological intervention) |
| 52. Dark urine and constipation | - None - Mild (Yellow urine, dry and hard stools requiring straining) - Moderate (Yellow urine with reduced volume, dry and hard stools, bowel movement every 2-3 days) - Severe (Yellow urine with reduced volume and painful, hard stools, bowel movement every 3 days or longer) |
| 53. Enlarged tongue with teeth marks | - None - Mild (Slightly enlarged tongue) - Moderate (Enlarged tongue with mild teeth marks) - Severe (Enlarged tongue with pronounced and extensive teeth marks) |
| 54. Pale tongue | - None - Mild (Mildly pale tongue) - Moderate (Pale-white tongue) - Severe (Extremely pale or blanched tongue) |
| 55. Red tongue with scant coating | - None - Mild (Slightly red tongue with scant coating) - Moderate (Red tongue with scant coating) - Severe (Purple-red tongue with no coating) |
| 56. Pale, enlarged tongue with a moist coating | - None - Mild (Pale, slightly enlarged tongue with moist coating) - Moderate (Pale, enlarged tongue with watery-smooth coating) - Severe (Pale, markedly enlarged tongue with an excessively moist coating, appearing to drip) |
| 57. Red tongue with yellow coating | - None - Mild (Dull-textured, red tongue with yellow coating) - Moderate (Dull-textured, deep red tongue with dry yellow coating) - Severe (Dull-textured, purplish red tongue with yellow-black coating) |
| 58. Yellow and greasy tongue coating | - None - Mild (Slightly yellow and greasy coating) - Moderate (Yellow and greasy coating) - Severe (Pronounced yellow and greasy coating) |
| 59. Purplish dark tongue with sublingual vein engorgement | - None - Mild (Dark red tongue with petechiae, slightly engorged bluish sublingual veins) - Moderate (Dark purple tongue with petechiae and ecchymosis, markedly engorged bluish-purple sublingual veins) - Severe (Bluish-purple tongue with severely engorged bluish-purple sublingual veins) |
| 60. Slippery and greasy tongue coating | - None - Mild (Slightly slippery and greasy) - Moderate (Slippery and greasy) - Severe (Markedly slippery and greasy) |
| 61. Weak and forceless pulse | - None - Mild (Slightly weak) - Moderate (Weak, unable to withstand pressure) - Severe (Profoundly weak and forceless) |
| 62. Thready and weak pulse | - None - Mild (Thready pulse) - Moderate (Thready and weak pulse) - Severe (Thready, rapid, and forceless pulse, or hollow pulse) |
| 63. Thready and rapid pulse | - None - Mild (Slightly thready and rapid pulse) - Moderate (Thready and rapid pulse) - Severe (Markedly thready and rapid pulse) |
| 64. Deep and slow pulse | - None - Mild (Slightly deep and slow pulse) - Moderate (Deep and slow pulse) - Severe (Markedly deep and slow pulse) |
| 65. Rapid pulse | - None - Mild (Slightly increased rate compared to baseline, 60-90 bpm) - Moderate (Significantly increased rate compared to baseline, 90-120 bpm) - Severe (Markedly increased rate compared to baseline, >120 bpm) |
| 66. Wiry pulse | - None - Mild (Slightly wiry) - Moderate (Wiry) - Severe (Markedly wiry) |
| 67. Wiry and slippery pulse | - None - Mild (Slightly wiry and slippery) - Moderate (Wiry and slippery) - Severe (Markedly wiry and slippery) |
| 68. Choppy or intermittent pulse | - None - Mild (Slightly choppy or intermittent) - Moderate (Choppy or intermittent) - Severe (Markedly choppy or intermittent) |
| 69. Slippery pulse | - None - Mild (Slightly slippery) - Moderate (Slippery) - Severe (Markedly slippery) |

**Table 2.** Missing data for variables

| **Variables** | **Missing (%)** |
| --- | --- |
| Basal metabolic rate, kcal | 3.17% |
| Fat-to-muscle mass ratio | 3.17% |
| Energy intake, kcal | 2.97% |
| DASH dietary pattern | 2.97% |
| Low-density lipoprotein, mmol/L | 1.78% |
| Glucose monitor frequency | 1.58% |

**Table 3.** SDQTM symptom frequency for T2DM with MetS

| **Variables** | **Frequency (%)** |
| --- | --- |
| Forgetfulness | 80.20 |
| Dry throat and mouth | 79.21 |
| Enlarged tongue with teeth marks | 76.04 |
| Lower back and knee weakness | 75.84 |
| Itching skin | 75.84 |
| Purplish dark tongue with sublingual vein engorgement | 72.87 |
| Dry mouth and bitter taste | 70.89 |
| Fatigue and weakness | 69.90 |
| Insomnia and profuse dreaming | 68.71 |
| Heavy sensation in the limbs | 68.71 |
| Frequent urination in large volume | 66.93 |
| Dark yellow or reddish urine | 65.94 |
| Dry eyes | 65.94 |
| Frequent nocturia | 62.18 |
| Hot flashes or night sweats | 59.21 |
| Numbness in the hands and feet | 58.42 |
| Abdominal distension after eating | 58.02 |
| Irritability or depression with frequent sighing | 57.43 |
| Squamous and dry skin | 55.64 |
| Short of breath and want of speech | 54.26 |
| Aversion to cold and cold limbs | 54.26 |
| Decreased libido | 52.87 |
| Thirst with desire for cold drinks | 52.87 |
| Spontaneous sweating | 51.49 |
| Blurred vision | 51.49 |
| Loose stools | 50.69 |
| Excessive eating and easy hunger | 45.54 |
| Puffy face and edema of the feet | 45.15 |
| Loose teeth and hair loss | 44.16 |
| Sticky, greasy sensation in the mouth | 44.16 |
| Pale, enlarged tongue with a moist coating | 43.76 |
| Dizziness | 42.97 |
| Fixed pain | 42.97 |
| Tinnitus and deafness | 42.77 |
| Palpitations | 42.18 |
| Yellow and greasy tongue coating | 41.39 |
| Distending pain in the abdomen | 40.59 |
| Sensation of heat in the palms, soles, and chest | 40.00 |
| Weak cough | 38.61 |
| Pale tongue | 38.22 |
| Dry hard stools | 37.03 |
| Weak and forceless pulse | 35.25 |
| Generalized edema | 35.25 |
| Difficulty with defecation, lack of strength | 35.05 |
| Slippery pulse | 33.47 |
| Lusterless nails | 32.67 |
| Susceptibility to common colds | 32.48 |
| Fullness in chest and hypochondrium | 29.90 |
| Wiry pulse | 28.71 |
| Reduced food intake and poor appetite | 28.51 |
| Dark urine and constipation | 28.12 |
| Petechiae on the skin | 27.92 |
| Deep and slow pulse | 26.93 |
| Cold pain in the abdomen | 26.93 |
| Rapid pulse | 26.93 |
| Slippery and greasy tongue coating | 25.94 |
| Pale lips and fingernails | 25.35 |
| Wiry and slippery pulse | 24.55 |
| Dusky or dull dark complexion | 24.16 |
| Purplish dark lips | 22.57 |
| Red tongue with yellow coating | 21.39 |
| Thready and weak pulse | 19.80 |
| Scanty urine | 16.83 |
| Thready and rapid pulse | 16.04 |
| Pale or sallow yellow complexion | 14.65 |
| Red tongue with scant coating | 13.27 |
| Nausea | 10.69 |
| Choppy or intermittent pulse | 4.16 |
| Vomiting | 1.19 |

**Table 4.** Fit statistics of different latent classes using the latent class analysis

| **Number of**  **classes** | **Sample size** | **AIC** | **BIC** | **adjusted BIC** | **Entropy** | **Estimated class population shares (%)** |
| --- | --- | --- | --- | --- | --- | --- |
| 3 | 505 | 43400.16 | 46931.89 | 44278.34 | 0.929 | 44.47, 28.42, 27.11 |
| 4 | 505 | 43452.78 | 48163.16 | 44624.04 | 0.937 | 23.86, 11.88, 22.96, 41.30 |
| 5 | 505 | 43698.04 | **49587.08** | **45162.38** | **0.948** | 16.90, 20.67, 38.82, 10.98, 12.63 |
| 6 | 505 | 44027.23 | **51094.92** | **45784.65** | **0.947** | 15.83, 16.00, 11.14, 18.69, 19.30, 19.04 |

Note: Bolded values indicate the criteria used to compare class number of 5 and 6 to determine the best model fit.

Table 5. Conditional probabilities for the 5-class latent class model

| **Class** | **Variable** | **0** | **2** | **4** | **6** |
| --- | --- | --- | --- | --- | --- |
| Class 1 | Fatigue and weakness | 0.07 | 0.82 | 0.10 | 0.01 |
|  | Short of breath and want of speech | 0.21 | 0.66 | 0.12 | 0.00 |
|  | Spontaneous sweating | 0.35 | 0.48 | 0.17 | 0.00 |
|  | Enlarged tongue with teeth marks | 0.31 | 0.35 | 0.31 | 0.03 |
|  | Weak and forceless pulse | 0.62 | 0.31 | 0.07 | 0.00 |
|  | Dizziness | 0.13 | 0.80 | 0.06 | 0.01 |
|  | Pale tongue | 0.67 | 0.31 | 0.01 | 0.00 |
|  | Dry throat and mouth | 0.00 | 0.67 | 0.32 | 0.01 |
|  | Sensation of heat in the palms, soles, and chest | 0.30 | 0.67 | 0.03 | 0.00 |
|  | Hot flashes or night sweats | 0.31 | 0.62 | 0.06 | 0.01 |
|  | Dark yellow or reddish urine | 0.14 | 0.80 | 0.05 | 0.00 |
|  | Dry hard stools | 0.32 | 0.44 | 0.19 | 0.05 |
|  | Aversion to cold and cold limbs | 0.12 | 0.79 | 0.08 | 0.01 |
|  | Puffy face and edema of the feet | 0.24 | 0.58 | 0.18 | 0.00 |
|  | Loose stools | 0.44 | 0.52 | 0.04 | 0.00 |
|  | Frequent urination in large volume | 0.15 | 0.71 | 0.14 | 0.00 |
|  | Pale, enlarged tongue with a moist coating | 0.63 | 0.33 | 0.04 | 0.00 |
|  | Weak cough | 0.30 | 0.62 | 0.08 | 0.00 |
|  | Susceptibility to common colds | 0.45 | 0.48 | 0.07 | 0.00 |
|  | Abdominal distension after eating | 0.14 | 0.67 | 0.20 | 0.00 |
|  | Difficulty with defecation, lack of strength | 0.27 | 0.51 | 0.20 | 0.01 |
|  | Lower back and knee weakness | 0.05 | 0.59 | 0.33 | 0.03 |
|  | Tinnitus and deafness | 0.29 | 0.53 | 0.12 | 0.06 |
|  | Loose teeth and hair loss | 0.26 | 0.57 | 0.12 | 0.04 |
|  | Decreased libido | 0.41 | 0.32 | 0.25 | 0.03 |
|  | Palpitations | 0.18 | 0.75 | 0.05 | 0.03 |
|  | Insomnia and profuse dreaming | 0.02 | 0.75 | 0.22 | 0.01 |
|  | Forgetfulness | 0.04 | 0.82 | 0.14 | 0.00 |
|  | Blurred vision | 0.17 | 0.70 | 0.12 | 0.01 |
|  | Lusterless nails | 0.33 | 0.53 | 0.12 | 0.01 |
|  | Numbness in the hands and feet | 0.11 | 0.79 | 0.06 | 0.04 |
|  | Dry eyes | 0.12 | 0.66 | 0.21 | 0.01 |
|  | Irritability or depression with frequent sighing | 0.13 | 0.81 | 0.06 | 0.00 |
|  | Frequent nocturia | 0.31 | 0.47 | 0.16 | 0.05 |
|  | Thirst with desire for cold drinks | 0.49 | 0.47 | 0.04 | 0.00 |
|  | Excessive eating and easy hunger | 0.37 | 0.57 | 0.06 | 0.00 |
|  | Distending pain in the abdomen | 0.19 | 0.74 | 0.07 | 0.00 |
|  | Generalized edema | 0.38 | 0.43 | 0.19 | 0.00 |
|  | Heavy sensation in the limbs | 0.00 | 0.86 | 0.13 | 0.02 |
|  | Dry mouth and bitter taste | 0.06 | 0.88 | 0.06 | 0.00 |
|  | Yellow and greasy tongue coating | 0.63 | 0.30 | 0.07 | / |
|  | Squamous and dry skin | 0.16 | 0.72 | 0.10 | 0.03 |
|  | Fixed pain | 0.38 | 0.56 | 0.06 | 0.00 |
|  | Purplish dark tongue with sublingual vein engorgement | 0.29 | 0.68 | 0.03 | 0.00 |
|  | Itching skin | 0.06 | 0.70 | 0.16 | 0.08 |
|  | Sticky, greasy sensation in the mouth | 0.32 | 0.65 | 0.03 | 0.00 |
|  | Slippery pulse | 0.76 | 0.19 | 0.05 | / |
| Class 2 | Fatigue and weakness | 0.07 | 0.72 | 0.21 | 0.00 |
|  | Short of breath and want of speech | 0.19 | 0.68 | 0.13 | 0.00 |
|  | Spontaneous sweating | 0.17 | 0.31 | 0.22 | 0.30 |
|  | Enlarged tongue with teeth marks | 0.21 | 0.37 | 0.31 | 0.11 |
|  | Weak and forceless pulse | 0.56 | 0.44 | 0.00 | 0.00 |
|  | Dizziness | 0.36 | 0.57 | 0.06 | 0.01 |
|  | Pale tongue | 0.64 | 0.22 | 0.12 | 0.01 |
|  | Dry throat and mouth | 0.07 | 0.46 | 0.44 | 0.03 |
|  | Sensation of heat in the palms, soles, and chest | 0.31 | 0.50 | 0.19 | 0.00 |
|  | Hot flashes or night sweats | 0.08 | 0.40 | 0.32 | 0.19 |
|  | Dark yellow or reddish urine | 0.23 | 0.74 | 0.03 | 0.00 |
|  | Dry hard stools | 0.80 | 0.16 | 0.03 | 0.01 |
|  | Aversion to cold and cold limbs | 0.50 | 0.40 | 0.10 | 0.00 |
|  | Puffy face and edema of the feet | 0.33 | 0.57 | 0.06 | 0.04 |
|  | Loose stools | 0.28 | 0.42 | 0.28 | 0.03 |
|  | Frequent urination in large volume | 0.30 | 0.57 | 0.13 | 0.00 |
|  | Pale, enlarged tongue with a moist coating | 0.52 | 0.43 | 0.05 | 0.00 |
|  | Weak cough | 0.56 | 0.43 | 0.01 | 0.00 |
|  | Susceptibility to common colds | 0.75 | 0.18 | 0.07 | 0.00 |
|  | Abdominal distension after eating | 0.34 | 0.53 | 0.12 | 0.00 |
|  | Difficulty with defecation, lack of strength | 0.73 | 0.25 | 0.01 | 0.00 |
|  | Lower back and knee weakness | 0.15 | 0.46 | 0.34 | 0.05 |
|  | Tinnitus and deafness | 0.62 | 0.33 | 0.04 | 0.00 |
|  | Loose teeth and hair loss | 0.63 | 0.27 | 0.06 | 0.04 |
|  | Decreased libido | 0.49 | 0.40 | 0.05 | 0.06 |
|  | Palpitations | 0.51 | 0.46 | 0.03 | 0.00 |
|  | Insomnia and profuse dreaming | 0.24 | 0.52 | 0.22 | 0.01 |
|  | Forgetfulness | 0.16 | 0.73 | 0.11 | 0.00 |
|  | Blurred vision | 0.39 | 0.50 | 0.11 | 0.00 |
|  | Lusterless nails | 0.68 | 0.22 | 0.10 | 0.00 |
|  | Numbness in the hands and feet | 0.18 | 0.62 | 0.18 | 0.03 |
|  | Dry eyes | 0.21 | 0.54 | 0.25 | 0.00 |
|  | Irritability or depression with frequent sighing | 0.19 | 0.61 | 0.13 | 0.06 |
|  | Frequent nocturia | 0.29 | 0.60 | 0.09 | 0.03 |
|  | Thirst with desire for cold drinks | 0.31 | 0.43 | 0.16 | 0.10 |
|  | Excessive eating and easy hunger | 0.44 | 0.46 | 0.09 | 0.01 |
|  | Distending pain in the abdomen | 0.49 | 0.48 | 0.03 | 0.00 |
|  | Generalized edema | 0.39 | 0.48 | 0.08 | 0.06 |
|  | Heavy sensation in the limbs | 0.08 | 0.70 | 0.21 | 0.01 |
|  | Dry mouth and bitter taste | 0.20 | 0.71 | 0.05 | 0.04 |
|  | Yellow and greasy tongue coating | 0.57 | 0.34 | 0.09 | / |
|  | Squamous and dry skin | 0.40 | 0.46 | 0.14 | 0.00 |
|  | Fixed pain | 0.45 | 0.42 | 0.09 | 0.04 |
|  | Purplish dark tongue with sublingual vein engorgement | 0.24 | 0.54 | 0.22 | 0.00 |
|  | Itching skin | 0.26 | 0.59 | 0.13 | 0.01 |
|  | Sticky, greasy sensation in the mouth | 0.48 | 0.52 | 0.00 | 0.00 |
|  | Slippery pulse | 0.71 | 0.22 | 0.07 | / |
| Class 3 | Fatigue and weakness | 0.74 | 0.25 | 0.01 | 0.00 |
|  | Short of breath and want of speech | 0.86 | 0.14 | 0.00 | 0.00 |
|  | Spontaneous sweating | 0.85 | 0.10 | 0.03 | 0.02 |
|  | Enlarged tongue with teeth marks | 0.20 | 0.35 | 0.36 | 0.08 |
|  | Weak and forceless pulse | 0.66 | 0.29 | 0.05 | 0.00 |
|  | Dizziness | 0.96 | 0.03 | 0.01 | 0.00 |
|  | Pale tongue | 0.61 | 0.35 | 0.03 | 0.01 |
|  | Dry throat and mouth | 0.61 | 0.33 | 0.06 | 0.00 |
|  | Sensation of heat in the palms, soles, and chest | 0.96 | 0.03 | 0.01 | 0.00 |
|  | Hot flashes or night sweats | 0.78 | 0.16 | 0.05 | 0.01 |
|  | Dark yellow or reddish urine | 0.59 | 0.41 | 0.00 | 0.00 |
|  | Dry hard stools | 0.85 | 0.11 | 0.04 | 0.00 |
|  | Aversion to cold and cold limbs | 0.70 | 0.22 | 0.09 | 0.00 |
|  | Puffy face and edema of the feet | 0.87 | 0.09 | 0.04 | 0.00 |
|  | Loose stools | 0.72 | 0.24 | 0.04 | 0.00 |
|  | Frequent urination in large volume | 0.51 | 0.42 | 0.07 | 0.00 |
|  | Pale, enlarged tongue with a moist coating | 0.55 | 0.33 | 0.12 | 0.00 |
|  | Weak cough | 0.87 | 0.13 | 0.00 | 0.00 |
|  | Susceptibility to common colds | 0.90 | 0.09 | 0.00 | 0.01 |
|  | Abdominal distension after eating | 0.78 | 0.22 | 0.00 | 0.01 |
|  | Difficulty with defecation, lack of strength | 0.88 | 0.11 | 0.01 | 0.00 |
|  | Lower back and knee weakness | 0.60 | 0.36 | 0.04 | 0.00 |
|  | Tinnitus and deafness | 0.80 | 0.18 | 0.01 | 0.01 |
|  | Loose teeth and hair loss | 0.78 | 0.20 | 0.03 | 0.00 |
|  | Decreased libido | 0.71 | 0.21 | 0.05 | 0.03 |
|  | Palpitations | 0.95 | 0.04 | 0.01 | 0.00 |
|  | Insomnia and profuse dreaming | 0.67 | 0.30 | 0.00 | 0.03 |
|  | Forgetfulness | 0.47 | 0.48 | 0.03 | 0.01 |
|  | Blurred vision | 0.82 | 0.16 | 0.02 | 0.00 |
|  | Lusterless nails | 0.92 | 0.08 | 0.00 | 0.00 |
|  | Numbness in the hands and feet | 0.77 | 0.19 | 0.01 | 0.03 |
|  | Dry eyes | 0.71 | 0.28 | 0.01 | 0.00 |
|  | Irritability or depression with frequent sighing | 0.85 | 0.15 | 0.00 | 0.00 |
|  | Frequent nocturia | 0.60 | 0.34 | 0.04 | 0.02 |
|  | Thirst with desire for cold drinks | 0.64 | 0.32 | 0.04 | 0.00 |
|  | Excessive eating and easy hunger | 0.80 | 0.18 | 0.02 | 0.00 |
|  | Distending pain in the abdomen | 0.97 | 0.03 | 0.00 | 0.00 |
|  | Generalized edema | 0.92 | 0.05 | 0.03 | 0.00 |
|  | Heavy sensation in the limbs | 0.81 | 0.18 | 0.00 | 0.01 |
|  | Dry mouth and bitter taste | 0.68 | 0.32 | 0.00 | 0.00 |
|  | Yellow and greasy tongue coating | 0.63 | 0.26 | 0.12 | / |
|  | Squamous and dry skin | 0.76 | 0.24 | 0.01 | 0.00 |
|  | Fixed pain | 0.88 | 0.11 | 0.01 | 0.00 |
|  | Purplish dark tongue with sublingual vein engorgement | 0.21 | 0.65 | 0.13 | 0.01 |
|  | Itching skin | 0.50 | 0.41 | 0.09 | 0.00 |
|  | Sticky, greasy sensation in the mouth | 0.89 | 0.10 | 0.01 | 0.00 |
|  | Slippery pulse | 0.63 | 0.29 | 0.08 | / |
| Class 4 | Fatigue and weakness | 0.32 | 0.61 | 0.07 | 0.00 |
|  | Short of breath and want of speech | 0.56 | 0.43 | 0.00 | 0.01 |
|  | Spontaneous sweating | 0.58 | 0.30 | 0.11 | 0.01 |
|  | Enlarged tongue with teeth marks | 0.26 | 0.33 | 0.37 | 0.04 |
|  | Weak and forceless pulse | 0.71 | 0.26 | 0.03 | 0.01 |
|  | Dizziness | 0.71 | 0.29 | 0.00 | 0.00 |
|  | Pale tongue | 0.59 | 0.38 | 0.03 | 0.00 |
|  | Dry throat and mouth | 0.18 | 0.69 | 0.13 | 0.00 |
|  | Sensation of heat in the palms, soles, and chest | 0.76 | 0.24 | 0.00 | 0.00 |
|  | Hot flashes or night sweats | 0.46 | 0.50 | 0.04 | 0.00 |
|  | Dark yellow or reddish urine | 0.42 | 0.57 | 0.01 | 0.00 |
|  | Dry hard stools | 0.63 | 0.27 | 0.07 | 0.03 |
|  | Aversion to cold and cold limbs | 0.48 | 0.42 | 0.09 | 0.02 |
|  | Puffy face and edema of the feet | 0.68 | 0.27 | 0.06 | 0.00 |
|  | Loose stools | 0.53 | 0.39 | 0.07 | 0.01 |
|  | Frequent urination in large volume | 0.38 | 0.51 | 0.11 | 0.00 |
|  | Pale, enlarged tongue with a moist coating | 0.56 | 0.41 | 0.02 | 0.01 |
|  | Weak cough | 0.71 | 0.27 | 0.01 | 0.01 |
|  | Susceptibility to common colds | 0.75 | 0.25 | 0.00 | 0.00 |
|  | Abdominal distension after eating | 0.46 | 0.50 | 0.04 | 0.00 |
|  | Difficulty with defecation, lack of strength | 0.73 | 0.23 | 0.03 | 0.01 |
|  | Lower back and knee weakness | 0.21 | 0.66 | 0.10 | 0.03 |
|  | Tinnitus and deafness | 0.63 | 0.28 | 0.06 | 0.04 |
|  | Loose teeth and hair loss | 0.63 | 0.31 | 0.05 | 0.01 |
|  | Decreased libido | 0.41 | 0.41 | 0.16 | 0.02 |
|  | Palpitations | 0.70 | 0.29 | 0.01 | 0.00 |
|  | Insomnia and profuse dreaming | 0.36 | 0.58 | 0.05 | 0.02 |
|  | Forgetfulness | 0.17 | 0.79 | 0.04 | 0.00 |
|  | Blurred vision | 0.54 | 0.42 | 0.04 | 0.01 |
|  | Lusterless nails | 0.77 | 0.20 | 0.03 | 0.00 |
|  | Numbness in the hands and feet | 0.53 | 0.42 | 0.03 | 0.02 |
|  | Dry eyes | 0.36 | 0.60 | 0.03 | 0.00 |
|  | Irritability or depression with frequent sighing | 0.51 | 0.42 | 0.07 | 0.00 |
|  | Frequent nocturia | 0.41 | 0.47 | 0.11 | 0.01 |
|  | Thirst with desire for cold drinks | 0.51 | 0.44 | 0.05 | 0.00 |
|  | Excessive eating and easy hunger | 0.63 | 0.36 | 0.01 | 0.00 |
|  | Distending pain in the abdomen | 0.71 | 0.28 | 0.01 | 0.00 |
|  | Generalized edema | 0.78 | 0.19 | 0.03 | 0.00 |
|  | Heavy sensation in the limbs | 0.34 | 0.63 | 0.02 | 0.01 |
|  | Dry mouth and bitter taste | 0.29 | 0.67 | 0.03 | 0.00 |
|  | Yellow and greasy tongue coating | 0.55 | 0.38 | 0.07 | / |
|  | Squamous and dry skin | 0.51 | 0.48 | 0.02 | 0.00 |
|  | Fixed pain | 0.62 | 0.36 | 0.02 | 0.00 |
|  | Purplish dark tongue with sublingual vein engorgement | 0.34 | 0.53 | 0.12 | 0.02 |
|  | Itching skin | 0.21 | 0.67 | 0.10 | 0.02 |
|  | Sticky, greasy sensation in the mouth | 0.63 | 0.34 | 0.03 | 0.00 |
|  | Slippery pulse | 0.62 | 0.26 | 0.11 | / |
| Class 5 | Fatigue and weakness | 0.04 | 0.44 | 0.42 | 0.09 |
|  | Short of breath and want of speech | 0.07 | 0.64 | 0.26 | 0.03 |
|  | Spontaneous sweating | 0.12 | 0.26 | 0.38 | 0.25 |
|  | Enlarged tongue with teeth marks | 0.19 | 0.31 | 0.40 | 0.10 |
|  | Weak and forceless pulse | 0.58 | 0.38 | 0.05 | 0.00 |
|  | Dizziness | 0.25 | 0.53 | 0.20 | 0.02 |
|  | Pale tongue | 0.61 | 0.30 | 0.09 | 0.00 |
|  | Dry throat and mouth | 0.01 | 0.12 | 0.53 | 0.34 |
|  | Sensation of heat in the palms, soles, and chest | 0.19 | 0.40 | 0.35 | 0.06 |
|  | Hot flashes or night sweats | 0.11 | 0.26 | 0.38 | 0.25 |
|  | Dark yellow or reddish urine | 0.06 | 0.69 | 0.24 | 0.02 |
|  | Dry hard stools | 0.42 | 0.33 | 0.20 | 0.04 |
|  | Aversion to cold and cold limbs | 0.33 | 0.34 | 0.28 | 0.05 |
|  | Puffy face and edema of the feet | 0.24 | 0.49 | 0.17 | 0.09 |
|  | Loose stools | 0.30 | 0.42 | 0.21 | 0.08 |
|  | Frequent urination in large volume | 0.12 | 0.37 | 0.38 | 0.14 |
|  | Pale, enlarged tongue with a moist coating | 0.55 | 0.37 | 0.08 | 0.00 |
|  | Weak cough | 0.33 | 0.46 | 0.18 | 0.03 |
|  | Susceptibility to common colds | 0.27 | 0.50 | 0.20 | 0.03 |
|  | Abdominal distension after eating | 0.11 | 0.44 | 0.34 | 0.11 |
|  | Difficulty with defecation, lack of strength | 0.36 | 0.36 | 0.26 | 0.03 |
|  | Lower back and knee weakness | 0.05 | 0.22 | 0.61 | 0.12 |
|  | Tinnitus and deafness | 0.31 | 0.45 | 0.14 | 0.11 |
|  | Loose teeth and hair loss | 0.24 | 0.46 | 0.19 | 0.11 |
|  | Decreased libido | 0.29 | 0.37 | 0.18 | 0.16 |
|  | Palpitations | 0.14 | 0.51 | 0.31 | 0.05 |
|  | Insomnia and profuse dreaming | 0.00 | 0.38 | 0.45 | 0.17 |
|  | Forgetfulness | 0.05 | 0.46 | 0.45 | 0.05 |
|  | Blurred vision | 0.23 | 0.44 | 0.29 | 0.05 |
|  | Lusterless nails | 0.37 | 0.47 | 0.15 | 0.02 |
|  | Numbness in the hands and feet | 0.11 | 0.39 | 0.36 | 0.14 |
|  | Dry eyes | 0.06 | 0.52 | 0.36 | 0.06 |
|  | Irritability or depression with frequent sighing | 0.06 | 0.40 | 0.47 | 0.07 |
|  | Frequent nocturia | 0.10 | 0.41 | 0.26 | 0.23 |
|  | Thirst with desire for cold drinks | 0.25 | 0.32 | 0.29 | 0.14 |
|  | Excessive eating and easy hunger | 0.19 | 0.52 | 0.28 | 0.02 |
|  | Distending pain in the abdomen | 0.22 | 0.65 | 0.12 | 0.02 |
|  | Generalized edema | 0.47 | 0.32 | 0.21 | 0.00 |
|  | Heavy sensation in the limbs | 0.02 | 0.41 | 0.45 | 0.12 |
|  | Dry mouth and bitter taste | 0.00 | 0.37 | 0.30 | 0.33 |
|  | Yellow and greasy tongue coating | 0.58 | 0.25 | 0.17 | / |
|  | Squamous and dry skin | 0.12 | 0.57 | 0.24 | 0.08 |
|  | Fixed pain | 0.27 | 0.37 | 0.25 | 0.11 |
|  | Purplish dark tongue with sublingual vein engorgement | 0.21 | 0.67 | 0.12 | 0.00 |
|  | Itching skin | 0.10 | 0.44 | 0.27 | 0.20 |
|  | Sticky, greasy sensation in the mouth | 0.16 | 0.64 | 0.15 | 0.05 |
|  | Slippery pulse | 0.68 | 0.16 | 0.16 | / |
